# Revealing the landscape of targeting mitochondrial functions and behaviors to overcome cancer chemoresistance

**DOI:** 10.1016/j.jncc.2025.02.007

**Published:** 2025-06-08

**Authors:** Haoyan Zhang, Sicheng Wang, Peng Wu, Zanmin Hu, Yani Chen, Yupeng Guan, Jun Pang

**Affiliations:** 1 Department of Urology, Kidney and Urology Center, Pelvic Floor disorders Center, The Seventh Affiliated Hospital, Sun Yat-Sen University, Shenzhen, China; 2 School of Medicine, Shenzhen Campus, Sun Yat-Sen University, Shenzhen, China; 3 Scientific Research Center, The Seventh Affiliated Hospital, Sun Yat-Sen University, Shenzhen, China

**Keywords:** Mitochondria, Cancer, Chemotherapy, Drug resistance

## Abstract

With the rapid progression of chemotherapies, the occurrence of chemoresistance is becoming a major obstacle in contemporary cancer treatment. As essential organelles, mitochondria perform diverse functions to provide ATP and various intermediates to modulate biosynthetic and bioenergetic processes, which are indispensable to cell survival. Recently, mitochondria have increasingly intrigued researchers for their unique influence on chemoresistance. This review explores the intricate relationship between mitochondria and chemoresistance. We delve into the complex roles that mitochondria play in chemoresistance, focusing on the aberrant alterations in mitochondrial behaviors and interactions with other organelles. We also review the subsequent impact of mitochondrial changes on cellular functions, such as metabolic reprogramming and the dysregulation of cell death. By presenting a retrospective analysis of previous research and elucidating the underlying mechanisms, we aim to reveal the potential of enhancing the efficacy of chemotherapies and overcoming cancer chemoresistance by targeting mitochondria. Hopefully, this review will provide directions for future research and the development of more viable drugs, ultimately improving the prognosis of cancer patients.

## 1 Introduction

Cancer chemoresistance has long been a significant barrier impeding the progress of cancer therapeutics. To date, two types of mechanisms have been elucidated regarding drug resistance. Intrinsic chemoresistance refers to the innate characteristic of cancer cells that hampers the effectiveness of treatment prior to its administration, which is due to preexisting resistance-mediating factors. On the other hand, acquired drug resistance is gained through adaptive response mechanisms to withstand the unfavorable environment caused by drug treatments. These adaptive mechanisms include the overexpression of drug extrusion channels, inhibition of cell death, metabolic reprogramming, and activation of alternative survival pathways.^1^

Recently, the pivotal role that mitochondria play in chemoresistance has been increasingly recognized.^2^ Mitochondria are the key organelles that perform diverse functions to provide Adenosine Triphosphate (ATP) and various intermediates, regulating biosynthetic and bioenergetic processes. Creating a dynamic as well as interconnected network, mitochondria are crucial to handle cell behavior. In cancer cells, their rapid response to environmental changes supports the maintenance of cellular homeostasis and significantly mitigates survival stress induced by chemotherapies. This adaptability largely contributes to metabolic reprogramming and ultimately leads to drug resistance.^3^^,^^4^

**Fig. 1:**
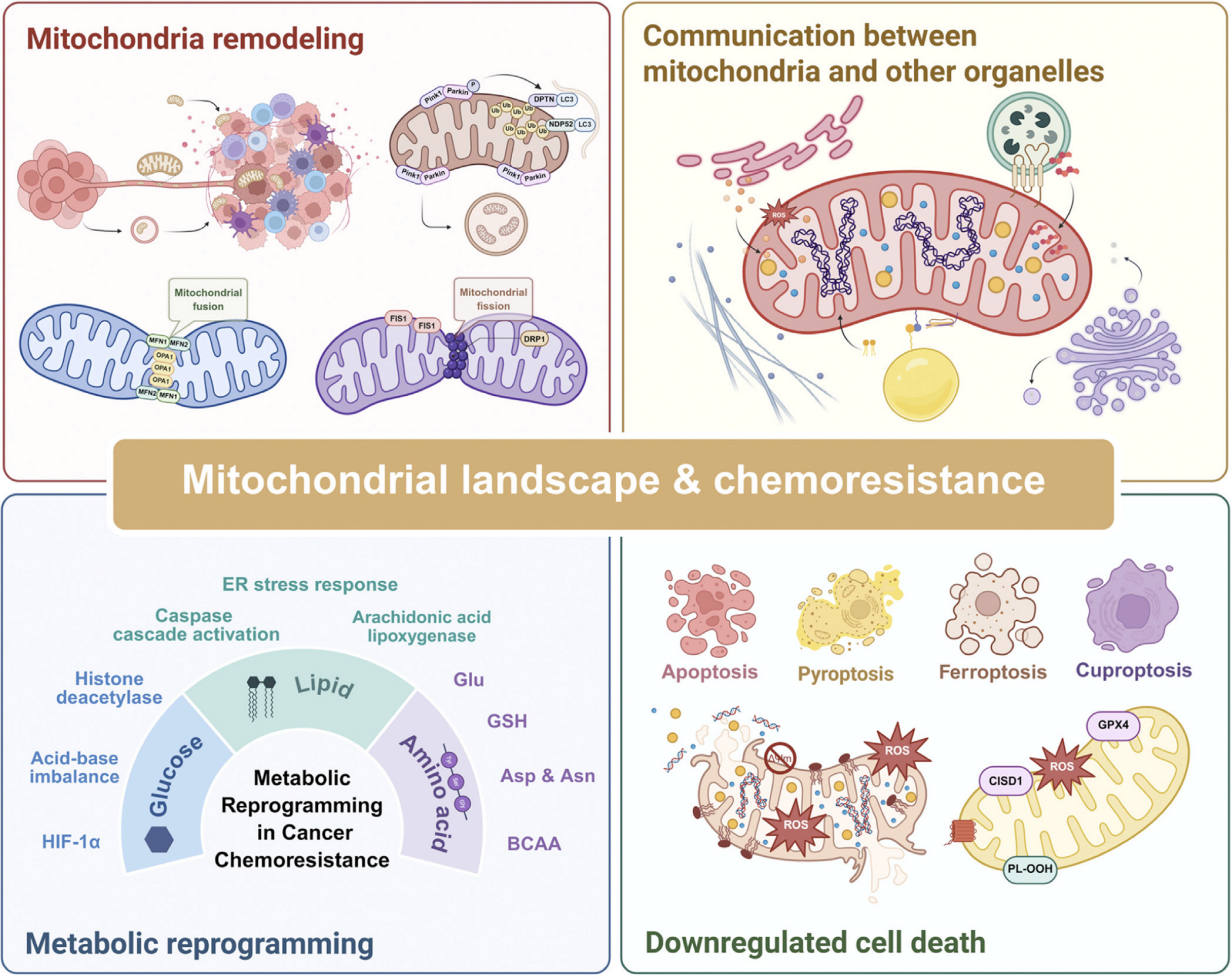
A brief overview of mitochondrial landscape and chemoresistance. This schematic illustrates the dynamic processes of mitochondrial remodeling, encompassing fusion and fission, and their interplay with other organelles in the context of cancer chemoresistance. Key pathways include ER stress responses, metabolic reprogramming, and modulation of cell death mechanisms (apoptosis, pyroptosis, ferroptosis, cuproptosis). The mitochondrial landscape influences chemoresistance through factors such as metabolic shifts, redox balance, and amino acid dynamics. Downregulated cell death pathways further contribute to therapeutic resistance. Asp, aspartate; Asn, asparagine; BCAA, branched-chain amino acid; CISD1, CDGSH iron sulfur domain 1; DRP1, dynamin-related protein 1; FIS1, mitochondrial fission protein 1; Glu, glucose; GPX4, glutathione peroxidase 4; GSH, glutathione; HIF-1α, hypoxia-inducible factor-1 alpha; MFN1/2, mitochondrial fusion proteins 1 and 2; OPA1, optic atrophy protein 1; PL-OOH, phospholipid hydroperoxide; ROS, reactive oxygen species.

In this review, we provide insights into the general mechanisms through which mitochondria generate chemoresistance in cancer cells, including abnormal mitochondrial changes, inter-organelle communication, and the mitochondria-induced inhibition of cell death. In short, our article aims to offer an overall understanding of the relationship between mitochondria and drug resistance and pave the way towards more efficient therapies (Fig. 1).

## 2 Mitochondria remodeling promotes cancer chemoresistance

Mitochondria in tumor cells undergo significant structural and functional remodeling. Through abnormal regulation of mitochondrial transfer, mitophagy, mitochondrial dynamics, and the acquisition of defective mitochondrial DNA, tumor cells modulate mitochondrial morphology and quantity, thereby influencing mitochondrial function. These adaptations enhance the tolerance of tumor cells to various survival pressures and contribute to the development of drug resistance.

### 2.1 Mitochondria transfer

**Fig. 2:**
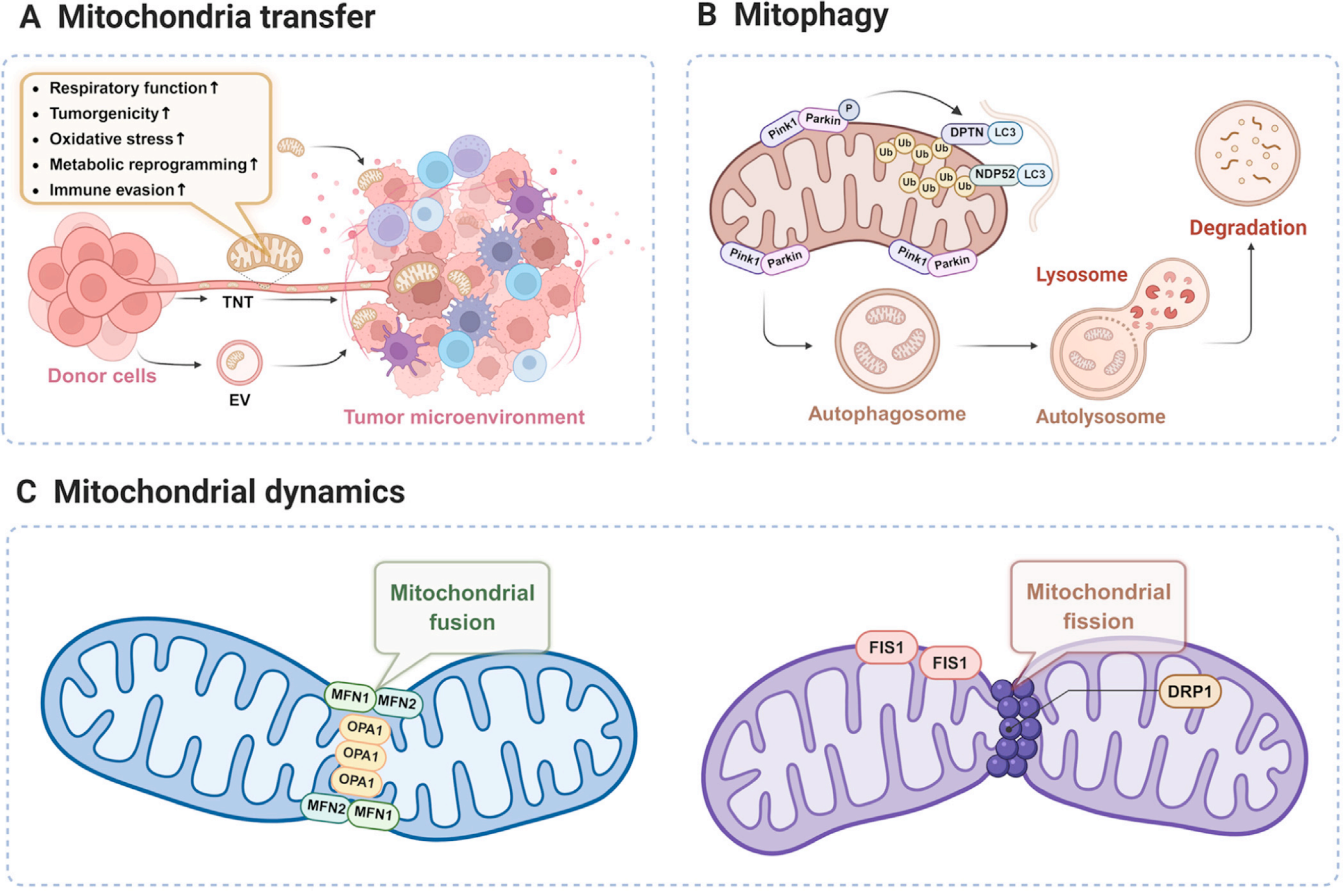
Mitochondria remodeling promotes cancer chemoresistance. Mitochondrial transfer is usually carried out through extracellular vehicles (EVs), tunnel nanotubes (TNTs) and free mitochondria in the captured cytoplasm. Mitophagy depends on extensive ubiquitination of mitochondrial surface proteins, with Pink1/Parkin as the main involved protein molecules. Mitochondrial fusion and fission play an important role in various cellular functions such as cell cycle, development and apoptosis, and contribute to chemotherapy resistance of cancer in many ways. DRP1, dynamin-related protein 1; FIS1, mitochondrial fission protein 1; LC3, Microtubule-associated protein 1 light chain 3; MFN1/2, mitochondrial fusion proteins 1 and 2; NDP52, nuclear dot protein 52 (CALCOCO2); OPA1, optic atrophy protein 1; Pink1, PTEN-induced putative kinase 1.

Mitochondria transfer is a newly unveiled form of intercellular communication, which involves the integration of either mitochondria genes or mitochondria themselves into a recipient cell.^19^ To date, several mechanisms of mitochondria transfer have been elucidated, among which extracellular vesicles (EVs), tunneling nanotubes (TNTs) and capture of free mitochondria are most comprehensively illustrated (Fig. 2A).^20^ As for cancer cells, mitochondria transfer within the tumor microenvironment is an important biological behavior commonly conducted via EVs and TNTs, and it contributes to cancer chemoresistance through multiple aspects (Table 1).^21^

**Table 1:** Mitochondrial dynamics in cancer chemoresistance.

Resistance mechanism	Cancer types	Resisted drug	Involved factors	Effects or involved mechanisms	References
Mitochondria transfer	T-ALL	Methotrexate	ICAM-1, TNTs	T-ALL cells adhere to MSCs through ICAM-1, forming TNTs that transfer mitochondria to MSCs, thereby reducing oxidative stress and leading to drug resistance.	^5^
	AML	Cytarabine	-	Treatment with Cytarabine enhances the capacity of leukemic blasts and leukemia initiating cells to endocytose mitochondria from bone marrow mesenchymal stromal cells, thereby intensifying drug resistance.	^6^
	GBM	Temozolomide	TNT, glutamine, orotic acid	Mesenchymal stem cells transfer mitochondria to GBM stem cells through TNTs, initiating metabolic reprogramming that includes a transition from glucose to glutamine and an increase in orotate turnover, thereby enhancing the resistance to temozolomide.	^7^
	OC	Cisplatin	CRL4, DRP1, Parkin/PINK1, AMPK, MFF	Upregulation of RL4 reduces DRP1 recruitment by inhibiting the phosphorylation of AMPK and MFF, which in turn decreases mitophagy through the Parkin/PINK1 pathway, thereby affecting the resistance of ovarian cancer to cisplatin.	^8^
Mitophagy	AML	Venetoclax	MFN2, OTUD5, Parkin/PINK1, TP53, BAX, BAK	Upregulation of MFN2 can enhance mitochondria-endoplasmic reticulum interactions and increase mitophagy flux, eliminating mitochondrial damage, thereby enhancing resistance to BH3 mimetics in AML.	^9^
	AML	Cytarabine	-	Devimistat, by impairing ATP synthesis, can be more effectively targeted by inhibiting mitochondrial fission and autophagy. The combination of Devimistat with Cytarabine and mitoxantrone may treat AML patients who are resistant to Cytarabine.	^10^
	BC	Paclitaxel	ATAD3A, PINK1, PD-L1	PINK1 can recruit PD-L1 to the mitochondria and degrade it through mitophagy. Paclitaxel disrupts the homeostasis of PD-L1 by increasing the expression of ATAD3A, thereby inhibiting PINK1-dependent autophagy.	^11^
	LUAD	Gefitinib	OPA1	Resistant lung adenocarcinoma cells upregulate OPA1, increase mitochondrial fusion, leading to elongated mitochondria, narrowed cristae, and enhanced ATP production, contributing to resistance against the tyrosine kinase inhibitor gefitinib.	^12^
Mitochondrial fusion	BC	Tamoxifen	MFN1, MFN2, OPA1, BAK	Depletion or pharmacological inhibition of MFN1 blocks mitochondrial fusion, restoring BAK oligomerization and cytochrome c release, thereby making resistant cells sensitive to apoptosis and enhancing the therapeutic efficacy of Tamoxifen.	^13^
	AML	Venetoclax	OPA1, BCL2, MCL1, CLPB	CLPB is upregulated in AML and further induced after venetoclax resistance. CLPB maintains mitochondrial cristae structure through interaction with OPA1, and its loss promotes apoptosis by inducing cristae remodeling and mitochondrial stress responses.	^14^
	BC	Paclitaxel	CD96, STAT3, OPA1	CD96 expression is associated with poor prognosis, and CD96 activation of the CD155-CD96-Src-Stat3-Opa1 pathway enhances mitochondrial fatty acid β-oxidation, thereby promoting chemoresistance in breast cancer stem cells.	^15^
	BC	Cisplatin	Spire1C, Arp2/3, DRP1, NRF2	The soft extracellular matrix activates DRP1-mediated mitochondrial fission, leading to an increase in mitochondrial ROS production, which in turn activates the NRF2 antioxidant pathway. Enhanced mitochondrial fission and antioxidant activity contribute to resistance against cisplatin.	^16^
Mitochondrial fission	HCC	-	DNML1 (DRP1), MFN1, TP53, NF-κB	Mitochondrial fission is increased through ROS-mediated AKT activation and subsequent coordinated regulation of the TP53 and NF-κB pathways, promoting autophagy and anti-apoptosis, which regulate the survival of HCC cells.	^17^
	NPC	Cisplatin	LMP1, AMPK, DRP1	EBV-encoded latent protein LMP1 potentiates mitochondrial fission, which is conducive to the survival and resistance of NPC cells by modulating the AMPK signal and the CDK1 signal to varying degrees.	^18^

Abbreviations: AML, acute myeloid leukemia; AMPK, adenosine 5‘-monophosphate-activated protein kinase; Arp2/3, actin-related protein 2/3; ATAD3A, ATPase family AAA-domain-containing protein 3; BAK, BCL-2 killer; BAX, BCL-2-associated X protein; BC, breast cancer; BCL-2, B-cell lymphoma-2; CD96, cluster of differentiation 96; CLPB, human caseinolytic peptidase B protein homolog; CRL4, Cullin-RING ligase 4; DNML1, dynamin-1-like protein; DRP1, dynamin-related protein 1; GBM, glioblastoma multiforme; HCC, hepatocellular carcinoma; ICAM-1, intercellular cell adhesion molecule-1; LMP1, latent membrane protein 1; LUAD, lung adenocarcinoma; MCL1, myeloid cell leukemia-1; MFF, mitochondrial fission factor; MFN, mitofusin; NF-κB, nuclear factor kappa-B; NRF2, nuclear factor erythroid 2-related factor 2; NPC, nasopharyngeal carcinoma; OC, ovarian cancer; OPA1, Optic atrophy 1; OTUD5, OTU deubiquitinase; PD-L1, programmed cell death ligand 1; PINK1, PTEN induced putative kinase 1; STAT3, signal transducer and activator of transcription 3; T-ALL, T-cell acute lymphoblastic leukemia; TP53, tumor protein 53; TNTs, tunnel nanotubes.

#### 2.1.1 Restoration of respiratory functions and tumorgenicity

Cancer cells with mitochondrial DNA (mtDNA) damage or depletion are deprived of aerobic respiration, often exhibiting low tumorigenicity and growth rates.^22^ However, studies has shown that mitochondrial DNA-deficient cancer cells are able to acquire whole mitochondria or mtDNA from neighboring cells to regain oxidative phosphorylation (OXPHOS), resulting in the restoration of respiratory functions as well as tumorigenic potential.^23^ This phenomenon was initially observed in mitochondria-deprived A549 lung cancer cells interacting with mesenchymal stem cells, and has since been widely reported in skin cancer cells, bladder cancer cells, hormone therapy-resistant metastatic breast cancer and a variety of other malignant tumors.^22^, ^23^, ^24^, ^25^ In breast cancer, this compensatory mechanism can promote chemotherapy-induced cancer stem cell-like cells to restore OXPHOS and metabolic activity, escape dormancy, and transition into a hormone therapy-resistant (HTR) phenotype.^25^ Such a compensatory mechanism helps cancer cells partially withstand mitochondrial damage, thereby increasing their resistance to mitochondria-targeted chemotherapies.

#### 2.1.2 Enhanced endurance of oxidative stress

Cancer cell apoptosis induced by enhancing intracellular reactive oxygen species (ROS) is an important mechanism of chemotherapeutic agents, and mitochondria are the most important producers of ROS.^26^ Therefore, upregulating mitochondrial ROS levels to kill cancer cells represents a viable strategy.^27^ Intriguingly, studies have revealed that cancer cells show resistance to oxidative stress through intercellular mitochondria transfer.^5^^,^^20^^,^^28^ Jurkat cells transfer their mitochondria to bone marrow mesenchymal stem cells (BM-MSCs) through tunneling nanotubes (TNTs) and vascular cell adhesion molecule-1 (VCAM-1). This process reduces intracellular aerobic oxidation levels and ROS production, thereby inducing resistance chemotherapeutic drugs that induce oxidative stress, such as ara-C or MTX.^5^ Under UV-induced stress, PC12 cells with mitochondrial damage become recipients of functional mitochondria from healthy PC12 cells and are rescued from apoptosis by sharing the oxidative burden with these donor cells.^28^ This enhanced endurance of oxidative stress ultimately results in resistance of relevant chemotherapy drugs.

#### 2.1.3 Reinforcement of metabolic reprogramming

Most tumors exhibit metabolic changes during cancer progression, which significantly contribute to environmental adaptation and resistance to treatment.^29^ Given the crucial role of mitochondria in biological processes, their transfer inevitably influences metabolic reprogramming, subsequently promoting chemoresistance. For cancer cells, the internalized mitochondria function as promoters of OXPHOS, producers of ATP, and metabolic fuel sources, generating high levels of glutamate, α-ketoglutarate, glutathione, nucleotides, and essential amino acids. This increases metabolic activity and strengthens antioxidant defenses in cancer cells.^6^^,^^30^ For example, acute myeloid leukemia (AML) cells can consume up to 16 mitochondria from BM-MSCs to enhance their mitochondrial biogenesis rate, maintain mitochondrial membrane potential (MMP), and thereby increasing replication potential and resistance to cytarabine.^6^ Glioblastoma stem cells induce metabolic reprogramming through mitochondrial transfer, promoting glutamine utilization and lactate turnover to gain resistance to temozolomide.^7^

#### 2.1.4 Promotion of immune evasion

Recent studies have provided a new perspective on tumor-T cell mitochondria transfer and immune evasion.^31^ Effector T cells and fibroblasts are major components of the tumor microenvironment. Fibroblasts release glutathione and cysteine, reducing platinum accumulation in the nucleus of ovarian cancer cells, while CD8^+^ T cells counteract this resistance mechanism by altering fibroblast metabolism. By hijacking mitochondria from immune cells via physical nanotubes, cancer cells enhance their metabolic capacity, meanwhile causing energy production shortages and dysfunction in T cells.^32^ The subsequential T cell depletion hampers chemotherapy drugs such as cisplatin from effecting and becomes a triggering factor for stromal-mediated chemoresistance.^33^^,^^34^

### 2.2 Mitophagy in cancer chemoresistance

A variety of mitochondrial quality control mechanisms have been discovered and proven to play a role in cancer progression and chemotherapy resistance to date, among which mitophagy, a specific type of organelle autophagy, is widely studied.^35^ Substantial evidence from previous research confirms that mitophagy is a crucial way to remove damaged mitochondria, thus sustaining cell homeostasis.^36^ Mitophagy primarily relies on the PINK1/Parkin pathway for the ubiquitination of mitochondrial surface proteins. When the mitochondrial membrane potential is compromised, the pathway for PINK1 to transfer to the inner mitochondrial membrane (IMM) is blocked, leading to the stable accumulation of PINK1 on the cytoplasmic side of the outer mitochondrial membrane (OMM). This accumulation subsequently recruits and activates Parkin. Once activated, Parkin functions as an E3 ubiquitin ligase to ubiquitinate proteins on mitochondria.^37^ Mitophagy can either support or mitigate chemoresistance, depending on the cellular contexts, as the accumulation of damaged mitochondria may increase oxidative stress, while the elimination of functionally impaired mitochondria helps enhance cellular vitality (Fig. 2B).^38^^,^^39^

Mitophagy can be carcinogenic in established cancer cell lines and enhances cancer stem cell survival.^39^^,^^40^ Moreover, recent studies indicate that mitophagy contributes to tumor chemoresistance as well.^10^^,^^35^ Regulation of mitophagy impacts cancer drug sensitivity, particularly when anti-mitochondrial drugs are applied.^41^ For instance, a study shows that loss of DDB1-CUL4-ROC1 E3 ubiquitin ligase (CRL4) could stimulate mitophagy to inhibit ovarian cancer cell proliferation. While inhibition of mitophagy partially reverses this disruption and even induces cisplatin resistance in OC.^8^ Nevertheless, increased mitophagy can promote drug resistance under certain conditions.

AML cells increase mitophagy under mitochondrial stress, resulting in a reduced number of mitochondria while maintaining normal respiration and metabolic capacity. This suggests that AML cells selectively retain healthier and more functional mitochondria via mitophagy, thereby enhancing prosurvival mechanisms and causing AML resistance to BH3-mimetics.^9^ It has also been shown that the tricarboxylic acid cycle inhibitor devimistat works better in the elderly patients with decreased mitophagy. Inhibition of mitophagy components also sensitizes cancer cells to devimistat.^10^ Additionally, it is worth noting that mitophagy not only profoundly affects chemotherapy resistance but also influences the efficacy of immunotherapy by recruiting PD-L1 into the mitochondria for degradation (Table 1).^11^

### 2.3 Mitochondrial dynamics in cancer chemoresistance

Reshaping mitochondrial structures is also a crucial mechanism for mitochondrial quality control.^42^ Through a host of recent studies, researchers have gradually realized that mitochondria, as highly dynamic organelles, are also actively involved in the regulation of cell signaling cascades.^42^^,^^43^ With continuous fusion and fission cycles, mitochondrial dynamics significantly affect the shape, size and position of mitochondria^43^^,^^44^. Mitochondrial fusion brings about a more interconnected mitochondrial network and dilutes accumulated mitochondrial DNA mutations and oxidized proteins. Correspondingly, mitochondrial fission can generate more discrete mitochondria capable of producing more ROS, thus promoting mitophagy or accelerating cell proliferation.^45^ In a physiological state, mitochondrial fusion and fission are mutually restrained, so that mitochondria can maintain a certain dynamic equilibrium. Once the balance is broken, it leads to impaired mitochondrial function and eventually cause diseases.^46^ Dysregulation of mitochondrial dynamics is strongly correlated with the tumor initiation and progression, suggesting that addressing mitochondrial dynamics may be a prospective approach to anticancer therapy.^47^

Mitochondrial fusion is often a defensive response. As mitochondria fuse their inner and outer membranes, their capability to resist stress is improved. It is induced by interactions between mitochondrial fusion proteins 1 and 2 (MFN1/2) of the OMM and optic atrophy protein 1 (OPA1) of the IMM (Fig. 2C). Mitochondrial fusion is responsible for the exchange of mtDNA, membrane phospholipids, respiration-related proteins and intermediates of mitochondrial tricarboxylic acid (TCA) cycle and is considered as an important factor for chemoresistance.^47^ For example, by enhancing mitochondrial oxidative metabolism and reducing sensitivity to cytochrome c release, the increase in OPA1 maintains the mitochondrial phenotype of gefitinib-resistant lung adenocarcinoma cells, enabling them to sustain oxidative metabolic capacity under gefitinib stress.^12^ Venetoclax treatment can lead to mitochondrial structural abnormalities and depolarization in AML cells, inducing tumor cell apoptosis. Studies have reported that the mitochondrial chaperone protein CLPB interacts with OPA1 to maintain the tight structure of mitochondrial cristae, reducing apoptosis induced by mitochondrial damage and thereby leading to Venetoclax resistance.^14^ Besides, the upregulation of MFN1 and increased interaction between MFN1 and MFN2 promote OMM fusion, which impedes the activation of BCL2-antagonist/killer 1 (BAK1) and inhibits cytochrome c release. This prevents tamoxifen-induced mitochondrial apoptosis and contributes to drug resistance.^13^

By contrast, mitochondrial fission transforms mitochondrial network into smaller organelles, promoting mitophagy to eliminate the depolarized mitochondria. Dynamin-related protein 1 (DRP1) is the key protein involved in mitochondrial fission, which is activated by phosphorylation and then recruited to OMM by interacting with DRP1 receptors. Upon recruitment to the mitochondrial surface, DRP1 assembles into a contractile ring, which encircles the mitochondrion and contracts to promote fission.^48^ This mechanism is actually analogous to certain motor proteins involved in endocytosis of the cell membrane (Fig. 2C).^49^ Research have proven that excessive mitochondrial fission and overexpression of the fission component mitochondrial fission protein 1 (FIS1) -DRP1 extensively participate in tumorigenesis, growth, metastasis and drug resistance.^50^ For instance, metastatic breast cancer cells can enhance their resistance to cisplatin by increasing DRP1-dependent mitochondrial fission, leading to increased production of ROS and activation of the nuclear factor erythroid 2-related factor 2 (NRF2) antioxidant transcriptional response.^16^ In hepatocellular carcinoma (HCC), upregulation of DRP1 leads to increased mitochondrial fission, which produces more ROS to activate mitophagy and directly inhibits mitochondria-dependent apoptosis. Moreover, it also promotes drug resistance through ROS-mediated activation of AKT to coordinate NF-κB and TP53 pathways to inhibit apoptosis.^17^ Certain viral products, such as EBV-encoded LMP1, can also activate mitochondrial fission within infected cells, affecting chemoresistance (Table 1).^18^

### 2.4 mtDNA defects

mtDNA defects in tumor cells, including mtDNA mutations, depletion, and copy number variations, has been demonstrated to induce drug resistance in various cancers. The pancreatic cancer cell line CFPAC-1 with a mutation in the ATP synthase 6 protein (ATPase 6) is resistant to cytochrome c-induced apoptosis and exhibits resistance to 5-fluorouracil (5-FU) and cisplatin.^51^ In the A549 non-small cell lung cancer cell line, a mutation in the mitochondrial complex I subunit gene MT-ND2, which encodes NADH dehydrogenase 2, leads to a 50% reduction in the NADH: ubiquinone oxidoreductase activity of the complex. This mutation results in compensatory upregulation of the nuclear coactivator peroxisome proliferator-activated receptor gamma coactivator-1 beta (PGC-1β), which promotes the development of a cisplatin-resistant phenotype.^52^ Recent studies have also shown that changes in mtDNA copy number are associated with tumor drug resistance. Reduced mtDNA copy number in esophageal squamous cell carcinoma (ESCC) patients can promote the depolarization of mitochondrial membrane potential (MMP) and induce epithelial-mesenchymal transition (EMT), leading to chemotherapy resistance.^53^ This alteration may be caused by abnormal D-loop replication.^54^ Moreover, mtDNA depletion in tumor cells has also been linked to malignant phenotypes and drug resistance. Suzuki et al. reported that mtDNA deletion renders human myeloid leukemia ML-1a cells resistant to TNF-induced apoptosis.^55^ MCF-7 breast cancer cells lacking mtDNA express EMT markers and exhibit resistance to hydroxytamoxifen.^56^ Li et al. found that PC-3 androgen-dependent prostate cancer cells lacking mtDNA possess high levels of cellular stemness, express a range of cancer stem cell markers (CD44^+^, ABCG2^+^), and are resistant to both chemotherapy and radiotherapy.^57^

## 3 Communication between mitochondria and other organelles influences cancer chemoresistance

Mitochondria are crucial hubs for the interaction with other organelles. They communicate with the endoplasmic reticulum, lipid droplets, lysosomes, Golgi apparatus, and the cytoskeleton through signal transduction, vesicle transport, and membrane contact sites. These organelles form a complex network that collectively regulates energy metabolism, biosynthesis, immune responses, and cell renewal. However, in tumors, abnormal organelle communication and dysregulation of downstream pathways can lead to severe mitochondrial dysfunction, which impairs mitochondrial regulation of energy metabolism and ion buffering, thereby promoting malignant transformation and the development of drug resistance in tumors.^58^

### 3.1 Mitochondria-endoplasmic reticulum contacts in cancer chemoresistance

**Fig. 3:**
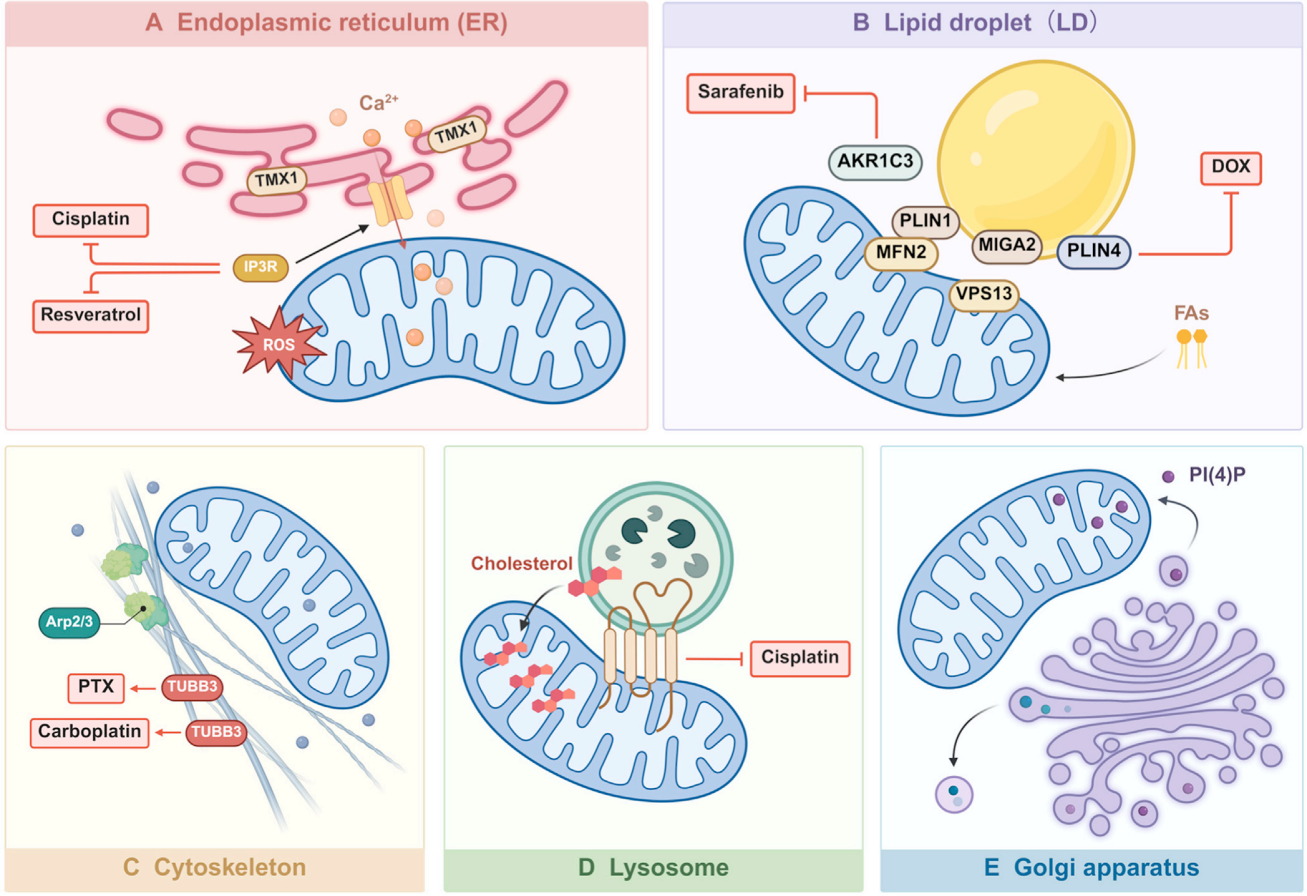
Interaction between mitochondria and various organelles. (A) Mitochondria interact with the ER through molecules such as TMX1 and IP3R, triggering biological changes such as Ca^2+^ transport, which lead to chemoresistance to Cisplatin in cancer cells. (B) Mitochondria interact with LD through molecules such as AKR1C3, PLIN1, and PLIN4, leading to chemoresistance to DOX in cancer cells. (C) Mitochondria are anchored to the cytoskeleton, and through interactions with the cytoskeleton, contribute to chemoresistance to PTX. (D) Mitochondria-lysosome contact mediates chemoresistance to Cisplatin. (E) Interactions between mitochondria and the Golgi apparatus have been observed and may represent potential targets for chemotherapy and overcoming chemoresistance. AKR1C3, aldo-keto reductase family 1 member C3; DOX, doxorubicin; DRP1, dynamin-related protein 1; ER, endoplasmic reticulum; FAs, fatty acids; IP3R, inositol 1,4,5-trisphosphate receptors; LD, lipid droplet; MFN2, mitochondrial fusion proteins 2; MIGA2, mitoguardin 2; PI(4)P, phosphatidylinositol 4-phosphate; PLIN1/4, perilipin 1/4; PTX, paclitaxel; ROS, reactive oxygen species; TUBB3, tubulin beta 3 class III; VPS13, vacuolar protein sorting 13.

Initially discovered in the early 1950s and biochemically isolated as mitochondria-associated endoplasmic reticulum (ER) membranes (MAMs) during the early 1990s, the mitochondria-endoplasmic reticulum contacts (MERCs) are now accepted as a platform that facilitates communication between the ER and the mitochondria and closely associates the two organelles.^59^ The MAM play a pivotal role in cell homeostasis and cell function regulation, making significant impacts on Ca^2+^ influx, lipid transfer, metabolism, autophagy and other biochemical processes, which implicates their affinity with cancer cells. The dysregulation of MAM is related to cancer cell metabolism, survival and sensitivity to cell death, subsequently generating resistance to chemotherapies.^60^^,^^61^ Targeting MAM may provide novel perspectives on overcoming chemoresistance in cancer cells (Fig. 3A).

#### 3.1.1 Dysfunction of MAM-mediated Ca^2+^ and ROS signaling in cancer cells

MAMs are pivotal microdomains for Ca^2+^ and ROS signaling in cancer cells.^62^ Multiple studies have shown that the dysfunction of several proteins located at the ER-mitochondria contact sites disrupts calcium homeostasis via directly remodeling Ca^2+^ flux and affecting ROS signals from ER to mitochondria, thereby mediating crucial cellular functions in tumors.^60^^,^^61^ In addition, ROS signals also interact with Ca^2+^ and participate in determining cancer cell fate.^63^

IP3Rs are regarded as one of the most important channel proteins that mediate calcium transport between ER and mitochondria.^64^ To facilitate ER–mitochondrial Ca^2+^ exchange, IP3Rs reside in a macromolecular complex, brought into close apposition to voltage-dependent anion channels (VDACs).^60^ The formation of a functional structure also relies on glucose-regulated protein of 75 kDa (GRP75), a key tether protein that closely connects IP3Rs and VDACs.^65^ Research have shown that in ovarian cancer cells, GRP75-facilitated MAM formation is enriched under cisplatin-exposure, which may contribute to the increase in [Ca^2+^]m and inhibit pro-apoptotic ROS accumulation, leading to cisplatin resistance.^66^ Additionally, the B-cell lymphoma 2 (Bcl-2) protein family is also found at MAMs, exerting anti-apoptotic functions by limiting ER-mitochondria Ca^2+^ transfer. It directly targets IP3Rs and prevents pro-apoptotic Ca^2+^ flux, bringing resistance to drugs such as cisplatin.^67^^,^^68^ Also, Bcl-2 homolog NRH/BCL-2L10 associates with and inhibits IP3R.^69^ Intriguingly, the outcome of function loss of another MAM resident PML resembles that of overexpression of BCL-2.^70^ In the absence of PML, IP3R-3 tends to be hyperphosphorylated, which in turn decrease ER-mitochondria Ca^2+^ flux to evade apoptosis.^71^

P53, the important tumor suppressor also localizes at MAM. It modulates the activity of sarco/ER Ca^2+^ ATPase (SERCA) and modulates ER-mitochondria cross-talk.^72^ In cells lacking p53, the downregulation of SERCA activity underlies cell-death resistance to chemotherapeutics. Moreover, under DNA damage, p53 activates the transcription of the ER-shaping proteins REEP1, REEP2 and EI24. This promotes MERC formation and interacts with VDAC2 to enhance ER-mitochondria Ca^2+^ transfer, leading to DNA damage-induced apoptosis.^73^ This may explain how DNA damaging drug resistance develops in p53-deficient cancer cells.

Several other mechanisms also account for dysregulation of Ca^2+^ at MAMs. For instance, FATE1, a cancer-testis antigen located at MAM, is able to uncouple the ER and mitochondria in adrenocortical carcinoma cells, thereby decreasing sensitivity to Ca^2+^-dependent apoptosis and to the chemotherapeutic drug mitotane.^74^ In addition, ROS signals also play a part. It is confirmed that downregulation of TMX1 and TMX3 induces oxidative stress in melanoma cells, with higher levels of MAM Ca^2+^ fluctuations and sensitivity to apoptosis.^75^ Furthermore, enriched VDAC1 in cisplatin-resistant ovarian cancer cells are reported to inhibit pro-apoptotic ROS accumulation and maintain cell survival.^66^

#### 3.1.2 MAM as autophagy activator in cancer cells

ER-mitochondria contact sites have also been found to be pivotal sites for autophagosome formation in mammalian cells.^76^ Accumulating evidence shows that the activation of autophagy desensitizes cancer cells to chemotherapy in various tumors. Claudie et al. revealed that MAM-mediated autophagy contributes to lipid catabolism, enhancing OXPHOS in acute myeloid leukemia cells, which promotes metabolic rewiring and supports cell survival under drug-induced stress.^77^ Another study by Missiroli et al. reported a correlation between PML deficiency and upregulated autophagy via AMPK/mTOR/ULK1 pathway, elucidating how the loss of PML from MAMs triggers chemoresistance in cancer cells.^78^ Furthermore, a recent study shows that mitochondrial chaperone Lon interacts with FUNDC1-ULK1 on MAMs, initiating mitophagy and thereby promoting hypoxia-induced chemoresistance in cancer therapy.^79^ Together, these studies demonstrate the tremendous potential of targeting MAM-related autophagy to overcome chemoresistance in cancer therapies.

#### 3.1.3 MAM as metabolic regulator in cancer cells

The role of metabolic reprogramming in drug resistance is being increasingly underlined, and targeting resident actors on MAM provides novel insights into metabolic regulation in cancer cells. For instance, reduced expression of the ER lumen protein thioredoxin TMX1 in cancer cells leads to decreased ER-mitochondria contact, inhibiting mitochondria-associated metabolism, lowering sensitivity to oxidative stress, and promoting growth while inhibiting apoptosis in cancer cells.^80^ Notably, changes in the lipid components of the ER and mitochondria in cancer cells can also lead to drug resistance.^81^ Jorida et al. found that reduced MAM in neuroblastoma leads to decreased ceramide transfer at the MERC, which reduces sensitivity to mitochondrial outer membrane permeability, decreasing mitochondria-associated apoptosis and inducing a multidrug-resistant phenotype.^82^

### 3.2 Mitochondria-lipid droplet contacts in cancer chemoresistance

Lipid droplet (LD) is a single-membrane organelle mainly found in adipose tissue cells, consisting of a neutral lipid core, including triglycerides (TAGs or TGs) and sterol esters, surrounded by a phospholipid monolayer composed of phosphatidylcholine (PC) and a wide range of proteins involved in lipid metabolism.^83^ LDs act as a lipid reservoir for energy production or membrane synthesis. Lipid storage in LDs is essential to protect cells from lipid toxicity.^84^

Fatty acid (FA) accumulates in LD in well-nourished cells, but during periods of nutrient deprivation, cells reprogram their metabolism from glycolysis to oxidation of FAs for ATP production, and the formation of contact sites between LD and mitochondria is increased during this process. FAs stored in tags in LD are transferred from LD to mitochondria.^85^ Several proteins are involved in the formation of LD-mitochondrial contact sites. For example, members of the SNARE protein family, such as SNAP23 and vesicle-associated membrane protein 4 (VAMP4), play roles in establishing the mitochondria-LD complex.^86^ The VPS13 family protein is recruited during starvation to LD-mitochondrial junctions, where it facilitates the transfer of FAs from LDs to mitochondria.^87^ The perilipin family protein perilipin1 (PLIN1) participates in LD-mitochondrial contact site formation in brown adipose tissue via interaction with MFN2.^88^ Similarly, perilipin5 (PLIN5) also mediates LD-mitochondrial interactions. Overexpression of PLIN5 inhibits lipolysis, increases LD accumulation, and stimulates mitochondrial lipid oxidation.^89^ In addition, Mitoguardin2 (MIGA2), a mitochondrial outer membrane protein, is also involved in the formation of LD-mitochondrial contact sites during the differentiation of white adipocytes (Fig. 3B).^90^

LD accumulation is frequently observed in many types of cancer and has become a new marker of human malignancy.^91^ In cancer cells, excess intracellular lipids are stored in LDs to prevent lipid toxicity and promote cell survival.^92^ Additionally, LD metabolism helps maintain membrane phospholipid homeostasis, thereby supporting the rapid proliferation of cancer cells.^93^ LD can also be used as an energy source to support tumor invasion and migration through β oxidation.^94^ Research has shown that doxorubicin-resistant triple-negative breast cancer (TNBC) cells (DOXO-R) exhibit reduced glycolysis, increased OXPHOS, and increased LD content in the cytoplasm, demonstrating a dependence on lipid oxidation. The most highly expressed protein in DOXO-R cells, perilipin 4 (PLIN4), protects the intracellular environment of TNBC cells from the toxic effects of excess fatty acids (FAs) and maintains the integrity of LDs. Knockout of the PLIN4 gene leads to significant cell death in DOXO-R cells, indicating a lipid-related vulnerability in chemotherapy-resistant TNBC.^95^ Additionally, studies have found that long-term treatment with sorafenib impairs fatty acid oxidation (FAO), leading to the accumulation of lipid droplets (LDs) in HCC cancer cells. High expression of the aldo-keto reductase AKR1C3 shifts the metabolic profile of HCC from FAO to glycolysis while inhibiting autophagy-dependent degradation of LDs, resulting in LD accumulation. This mechanism is essential for mitigating sorafenib-induced mitochondrial lipotoxicity and dysfunction. Reducing AKR1C3 expression drives the metabolic shift in HCC cells from glycolysis back to FAO, thereby limiting chemotherapy resistance in HCC.^96^ The expression of progesterone receptor membrane component 1 (PGRMC1), a heme-binding protein, was found to be increased in paclitaxel-tolerant cancer cells (PCC), contributing to the colocalization of LD and mitochondria and the promotion of FAO, resulting in more accumulating mitochondrial ROS. However, activating PGRMC1 to induce ferroptosis may also be a potential strategy for eliminating chemotherapy-resistant cancer cells.^97^ In summary, these relevant studies have shown the role of LD and mitochondrial HU interaction in mediating chemotherapy resistance. Targeting LD-mitochondria contact sites may provide novel therapeutic approaches for overcoming tumor chemoresistance.

### 3.3 Interactions between mitochondria and other organelles

Mitochondria-lysosome contact sites (MLCSs) is a newly discovered form of interaction between the two membranous organelles, which has been reported to mediate mitochondrial dynamics, regulate the transfer of lipid, calcium or iron and dynamically interact with other organelles.^98^ Several studies have disclosed that MLCSs support cancer cell survival, indicating their role in chemoresistance.^98^ In HCC, TM4SF5-enriched MLCSs mediate glucose catabolism via facilitating cholesterol export for mitochondrial reprogramming, promoting cancer cell biosynthesis.^99^ Additionally, MLCSs may be upregulated under hypoxic conditions, reinforcing the cleavage of VDAC1 to resist survival stress.^100^ The pivotal regulator of MLCSs, RAB7 GTPase, whose expression is mediated by mitochondria-derived vesicles, has also been proved to play a critical role in response to cisplatin resistance.^101^ Besides, targeting other ways of mitochondria-lysosome crosstalk to overcome cancer chemoresistance has attracted increasing interest. Via interfering mitochondria-lysosome communication, cancer cells can be sensitized to chemotherapies through alterations in mitophagy, lipid metabolism and cell signaling (Fig. 3D).^102^, ^103^, ^104^

Interaction between mitochondria and the cytoskeleton are also indispensable for mitochondria functions, affecting mitochondrial mobility, morphology and distribution, subsequently influencing cancer cell behaviors.^105^ The cytoskeleton network plays an essential part in regulating mitochondrial rearrangement, which is closely linked to OXPHOS.^106^ In addition, the cytoskeleton-mediated mitochondria relocation contributes to EMT, while mitochondria, in turn, modulate actin activity to mediate cancer cell migration.^107^ The Arp2/3 complex is indispensable in the process of actin cytoskeleton assembly and is also involved in the interaction between the cytoskeleton and mitochondria.^108^ Literature reports suggest that soft matrices can induce increased ER-Ca^2+^ extrusion in breast cancer, thereby increasing actin filaments around mitochondria, and subsequently recruit DRP1 to promote mitochondrial fission. Inhibiting the Arp2/3 complex reduces the interaction between DRP1 and mitochondria, thereby disrupting mitochondrial division.^109^ Another important player participating in mitochondria-cytoskeleton interactions is the VDACs.^110^ It has also been discovered that β-tubulin, the regulator of VDACs, is implicated in altered energy metabolism, acquired resistance, and cross‑resistance in cancer cells, underscoring its role in chemoresistance (Fig. 3C).^110^^,^^111^

Golgi apparatus is a critical factory where proteins from ER are further processed, packaged and transported to their final destinations. Previous studies have revealed several ways through which mitochondria correlates with the Golgi apparatus. For instance, the Golgi-derived PI(4)P-containing vesicles have been found to assist in mitochondria fission, and stable Golgi-mitochondria complexes may be relevant with the formation of Golgi Ca^2+^ gradients in pancreatic acinus cells (Fig. 3E).^112^, ^113^, ^114^ However, to estimate their potential in cancer therapies, further research needs to be done.

## 4 The roles of metabolic reprogramming in cancer chemoresistance

Tumor cells undergo metabolic reprogramming through the regulation of mitochondrial function to meet the energy demands and biosynthetic requirements associated with rapid proliferation. Alterations in the metabolism of key substrates—glucose, lipids, and amino acids—within mitochondria contribute to enhanced drug resistance in tumor cells. These metabolic adaptations modulate the intracellular environment, mitigate oxidative stress, and augment energy production, collectively representing a critical mechanism underlying the acquisition of drug resistance in cancer.

### 4.1 Glucose metabolic reprogramming in cancer chemoresistance

A major form of metabolic reprogramming in cancer is aberrant glucose metabolism.^137^ In the early 1920s, Otto Warburg proposed the famous “Warburg effect”, which suggests that instead of mitochondrial OXPHOS, cancer cells prefer glycolysis for energy production, even under oxygen-rich conditions.^138^ Previous studies have revealed that the acquisition of chemoresistance is linked to the dysregulation of glucose metabolism and glycolysis.^139^ The following are the most intensively studied mechanisms through which cancer cells rewire glucose metabolism and acquire chemoresistance (Table 2).

**Table 2:** The roles of metabolic reprogramming in cancer chemoresistance.

Remodeled metabolism	Mechanism	Involved factors	Consequential alteration	Effects on tumors	References
Glucose	Decreases influx of pyruvate into mitochondria.	Pyruvate	Reduces the level of intracellular ROS.	Induces resistance to apoptosis and DNA damage.	^115^
	Promotes overexpression of PFKFB3 in HCC.	PFKFB3, AKT, ERCC1	Promotes glycolysis and evasion of G2/M DNA damage checkpoint.	Promotes cell proliferation and DNA repair.	^116^
	Lacks ATP and CO2.	—	Establishes intracellular alkaline pH and extracellular acidity.	Repels the intake of weakly basic anti-cancer drugs and causes MDR.	^117^ ^118^ ^119^
	Increases glucose metabolism in TAMs.	Cathepsin B, O-GlcNAc	Promotes O-GlcNAcylation of lysosomal Cathepsin B.	Promotes cancer metastasis and chemoresistance.	^120^
	Elevates expression of AKR1B10 in lung cancer BM cells.	AKR1B10, LDHA, CCNB1	Promotes glycolysis and accelerated DNA replication and cell cycle.	Reduces sensitivity to PEM.	^121^
	Aberrantly activates PI3K/Akt and Wnt/β-catenin signaling in CRC.	HIF-1α	Increases HIF-1α expression.	Induces resistance to 5-fluorouracil.	^122^
Lipid	Upregulates the level of CD36 in HER2+ breast cancer.	CD36	Upregulates fatty acid uptake.	Induces resistance to anti-HER2 treatments.	^123^
	Elevates phospholipids synthesis with DAG as a precursor.	DAG	Alters membrane lipid composition and the production of lipid second messengers.	Promotes tumor metastasis and drug resistance.	^81^
	Promotes accumulation of LD.	LPC, AKR1C3, LPCAT2	Impairs caspase cascade activation and endoplasmic reticulum stress response.	Induces resistance to 5-fluorouracil and oxaliplatin therapy.	^124^ ^96^ ^91^
	Upregulates the expression of STAT3 and FNR-related genes in gastric cancer cells.	STAT3	Negatively regulates ferroptosis.	Promotes tumor progression and chemoresistance.	^125^
	Upregulates the expression of MTTP in in exosomes of colorectal cancer patients with a high body-fat ratio.	MTTP	Decreases the ratio of polyunsaturated fatty acids and lipid ROS levels.	Promotes tumor progression and chemoresistance.	^126^
	Elevates the expression of lncROPM in BCSCs.	lncROPM, PLA2G16	Upregulates PLA2G16 expression, phospholipid metabolism and free fatty acid production.	Maintains BCSC stemness and promotes chemoresistance.	^127^
Amino acid	Augments glutamine metabolism.	Glutamine	Increases oxygen consumption in OXPHOS.	Promotes hypoxia-induced chemoresistance.	^128^
	Upregulates amphiregulin expression in human chondrosarcoma.	Amphiregulin	Promotes NADPH production and inhibited ROS accumulation.	Enhances glutamine metabolism and cisplatin resistance.	^129^
	Glutamine metabolism activates mTORC1 via alpha-ketoglutarate-dependent pathway; and a secondary ATP/AMPK-dependent pathway.	α-ketoglutarate, mTORC1, AMPK	Inhibits autophagy.	Promotes cell growth and enhances chemoresistance.	^130^
	Constitutively produces and releases asparagine through mass action.	GCN2, ATF4	Restores tumor growth in the context of respiration impairment.	Generates resistance to mitochondrial inhibitors.	^131^
	Amplifies the number of asparagine synthetase copies.	Asparagine synthetase	Increases resistance to oxidative stress.	Promotes the ability to proliferate and spread.	^132^
	Enhances OXPHOS and increases Asp generation.	Aspartate	Interrupts NAD+/NADH homeostasis and inhibits aspartate biosynthesis.	Generates resistance to EGFR inhibitors.	^133^
	Leucine supplementation inhibits autophagy via mTOR.	Leucine, mTOR	Sensitizes AML to DOX.	AML responds better to doxorubicin treatment.	^134^
	LLGL2 regulates leucine transporter SLC7A5.	LLGL2, SLC7A5, leucine	Breast cancer develops resistance to tamoxifen.	Enhances chemoresistance.	^135^
	Notch signaling increases valine-transporting tRNA levels.	Valine, mitochondrial complex I	Mitochondrial complex I synthesis is inhibited in T-ALL.	Mediates the progression of T-ALL.	^136^
	Doxorubicin loaded with leucine plymer induces mTOR activation.	Leucine	Inhibits autophagy.	Enhances robust apoptosis and chemosensitivity in AML.	^134^

Abbreviations: AKR1B10, aldo-keto reductase 1B10; AKR1C3, aldo-keto reductase 1C3; AKT, alpha serine/threonine-protein kinase; AML, acute myeloid leukemia; AMPK, adenosine 5′-monophosphate-activated protein kinase; ATF4, activating transcription factor 4; BCSCs, breast cancer stem cells; CCNB1, cyclin B1; CD36, cluster of differentiation 36; DAG, diacylglycerol; DOX, doxorubicin; EGFR, epidermal growth factor receptor; ERCC1, excision repair cross complementing group 1; FNR, ferredoxin-NADP+ reductase; GCN2, general control nonderepressible 2; HER2, human epidermal growth factor receptor 2; HIF-1α, hypoxia inducible factor-1; LD, lipid droplet; LDHA, lactate dehydrogenase; LLGL2, LLGL scribble cell polarity complex component 2; lncROPM, long noncoding RNA ROPM; LPC, lysophosphatidyl choline; LPCAT2, lysophosphatidylcholine acyltransferase 2; mTORC1, mechanistic target of rapamycin complex 1; MTTP, microsomal triglyceride transfer protein; NADPH, nicotinamide adenine dinucleotide phosphate; O-GlcNAc, O-GlcNAcylation; OXPHOS, oxidative phosphorylation; PEM, pembrolizumab; PFKFB3, 6-phosphofructo-2-kinase/fructose-2,6-biphosphatase 3; PLA2G16, adipose phospholipase A2; ROS, reactive oxygen species; SLC7A5, amino acid transporter LAT1; STAT3, signal transducer and activator of transcription 3; T-ALL, T-cell acute lymphoblastic leukemia.

#### 4.1.1 Reinforcement of DNA repair and endurance of oxidative stress

DNA replication is a vital target for chemotherapies. When this essential process is disrupted, cancer cells undergo apoptosis or cell death. However, aberrant glucose metabolism can strengthen DNA repair system, subsequently promoting drug resistance and mitigating DNA damage.^140^ Elevated levels of ROS are an important cause of DNA damage and genomic instability. Glucose metabolic reprogramming provides an alteration from oxidative to reductive metabolism, which downregulates the activity of mitochondria, thereby reducing the production of ROS.^115^ In breast cancer, there can even be a restriction on pyruvate entering the mitochondria due to the upregulation of pyruvate dehydrogenase kinase 4 (PDK4).^141^ The glycolytic enzymes and intermediates also relieve oxidative stress, promote pentose phosphate pathway and therefore maintain cellular redox balance.^139^ In contrast, the loss of GLUT1 and GPI1, which induces metabolic rewiring toward OXPHOS and ROS accumulation, sensitizes cancer cells to TNF-α-induced cell death.^142^ Besides, the mutation in glucose metabolic rewiring-related genes can also enhance chemoresistance by reinforcing DNA repair.

DNA repair resulting from glucose metabolic reprogramming involves multiple mechanisms. The altered gene PFKFB3 enhances the capacity for DNA repair during glycolysis via the PFKFB3/Akt pathway by increasing the expression of ERCC1, a 5′-3′ structure-specific endonuclease, thereby promoting DNA repair and ultimately leading to the failure of chemotherapy in HCC.^116^ Elevated lactate levels, resulting from aberrantly activated glycolysis, can enhance DNA repair and promote cisplatin resistance in cervical cancer cells through the inactivation of histone deacetylase.^143^ Recent reports have also shown that lactate can drive the lactylation of Nijmegen breakage syndrome protein 1 (NBS1) at lysine 338, promoting homologous recombination (HR)-mediated DNA repair.^144^

#### 4.1.2 Dysregulation of pH dynamics

A decrease in ATP and CO_2_ production, resulting from glucose metabolic reprogramming significantly influences pH dynamics in cancer cells, as they are indispensable intracellular sources of H^+^.^145^ This reduction in ATP and CO₂ also activates membrane pumps that establish intracellular alkaline pH and extracellular acidity.^117^ The inverted pH gradient contributes to multidrug resistance (MDR) by repelling the intake of weakly basic anticancer drugs, such as mitoxantrone and anthracyclines doxorubicin.^118^^,^^119^ Additionally, the extracellular acidity upregulates functional membrane efflux pumps that accelerate the export of anticancer drugs, further contributing to MDR.^145^

#### 4.1.3 Other mechanisms of chemoresistance induced by glucose metabolic rewiring

Aberrant activation or inactivation of signaling pathways and cell cycle-related genes is associated with chemoresistance during metabolic reprogramming. AKR1B10 inhibits the sensitivity of lung cancer brain metastatic subpopulation cells to pemetrexed (PEM) by regulating lactate dehydrogenase (LDHA) expression and increasing lactate-promoted glycolysis. This process activates the transcription of the cell cycle-related gene CCNB1, thereby promoting cell survival.^146^ Additionally, upregulated HIF-1α levels, mediated by the aberrant activation of PI3K/Akt and Wnt/β-catenin signaling, induce resistance to 5-fluorouracil (5-FU) in colorectal cancer (CRC).^147^ Other mechanisms include exosome-induced cancer stem cell formation, metabolic dysfunction-mediated upregulation of autophagy. However, many aspects of these processes remain to be explored.^137^^,^^140^

### 4.2 Lipid metabolic reprogramming in cancer chemoresistance

**Fig. 4:**
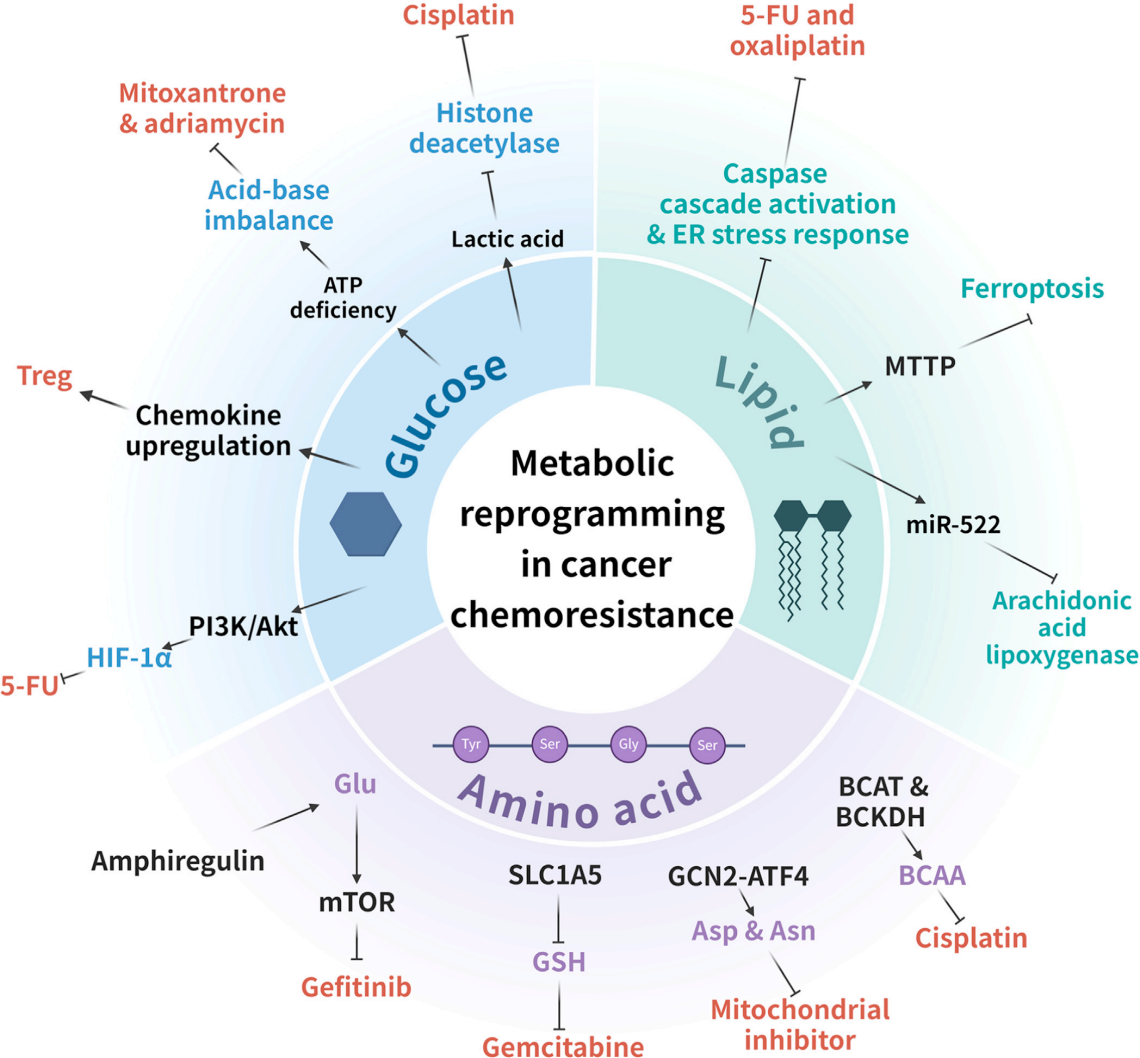
Cellular physiological changes caused by mitochondrial dysfunction enhance chemotherapy resistance in cancer. The metabolic reprogramming processes associated with mitochondria have different roles in cancer chemotherapy resistance, shown here as important molecules in glucose, lipid and amino acid metabolic reprogramming and related drugs. 5-FU, 5-fluorouracil; Asn, asparagine; Asp, aspartate; BCAA, branched-chain amino acid; BCAT, branched-chain amino acid transaminase; BCKDH, branched-chain alpha-ketoacid dehydrogenase; ER, endoplasmic reticulum; GCN2-ATF4, general control nonderepressible 2–activating transcription factor 4; Glu, glucose; GSH, glutathione; HIF-1α, hypoxia-inducible factor-1 alpha; miR-522, microRNA-522; MTTP, microsomal triglyceride transfer protein; SLC1A5, solute carrier family 1 member 5; Treg, regulatory T cells.

Lipid metabolic reprogramming is a novel target for overcoming tumor chemoresistance. Cancer cells undergo lipid metabolic reprogramming to adapt to the tumor microenvironment, which encompasses a sequence of complex procedures, including lipid uptake, synthesis, storage, and oxidation (Fig. 4).^148^

Cancer cells exhibit a propensity to ingest lipids to support biosynthesis, energy production, and fat storage. The requirement is met by increasing lipid synthesis precursors through both endogenous and exogenous pathways.^148^ Exogenous lipids involve the participation of transport molecules, including the cell surface protein CD36, the family of fatty acid transport proteins (FATPs), and fatty acid-binding proteins (FABPs).^149^ The upregulation of CD36 in breast cancer has been shown to be associated with resistance to HER2-targeted therapy.^150^ To respond to the high consumption of lipids, cancer cells also increase the content of endogenous lipids, which are mainly produced by de novo lipogenesis (DNL) using acetyl coenzyme A as a substrate. Diacylglycerol (DAG) serves as an intermediate in triacylglycerol synthesis from long-chain fatty acid derived from DNL. Phospholipid synthesis using DAG as a precursor is elevated in cancer and plays a role in regulating tumor metastasis and drug resistance by modifying membrane lipid composition and generating lipid second messengers.^151^ LDs are essential lipid-storing organelles conserving unsaturated FAs. A crucial function of LD is to reduce lipid peroxidation and relieve oxidative pressure in cancer cells. Accumulation of LD in cancer cells impairs caspase cascade activation and ER stress response, which can lead to resistance to 5-FU and oxaliplatin therapy.^96^^,^^152^^,^^153^

Remodeled lipid oxidation impacts cancer cell chemoresistance in multiple aspects. The accumulation of lipid peroxides in cancer cells can elevate intracellular ROS levels and sensitize cancer cells to ferroptosis.^154^ 5-FU-resistant gastric cancer cells exhibit upregulation of Src-signal transducer and activator of transcription 3 (STAT3) and ferroptosis negative regulation-related genes. Genetic inhibition of STAT3 activity has been demonstrated to trigger ferroptosis through lipid peroxidation and Fe^2+^ accumulation in gastric cancer cells, subsequently alleviating chemoresistance.^155^ In tumors, exosomes play an intriguing role in resistance to oxidative stress under lipid metabolic reprogramming. Upregulation of microsomal triglyceride transfer protein (MTTP) expression in exosomes of CRC patients with a high body-fat ratio leads to a decreased ratio of polyunsaturated fatty acids and lipid ROS levels, thus suppressing ferroptosis and reducing sensitivity to chemotherapies.^126^ The cancer-associated fibroblasts in TME produce exosomes containing miR-522, which inhibits arachidonic acid lipoxygenase, thereby reducing lipid-ROS accumulation in cancer cells, ultimately leading to reduced chemosensitivity.^156^ Abnormal lipid oxidation in cancer stem cells (CSCs) also plays a part in chemoresistance. CSCs in primary breast cancer maintain stemness and chemoresistance by reducing intracellular ceramide abundance through high expression of the lipid transporter ABCA12.^157^ LncROPM, highly expressed in CSCs, upregulates PLA2G16 expression and significantly promotes phospholipid metabolism and free fatty acid production. This activation stimulates PI3K/AKT, Wnt/β-catenin, and Hippo/YAP signaling to maintain CSC stemness and promotes chemoresistance.^127^ CSCs also regulate fatty acid β-oxidation via JAK/STAT3 signaling, enhancing self-renewal and chemoresistance (Table 2).^158^

### 4.3 Amino acid metabolic reprogramming in cancer chemoresistance

Amino acid metabolic reprogramming is an important aspect of tumor metabolic reprogramming. Abnormalities in the rate of amino acid uptake, metabolic pathways, metabolites, or key metabolic enzymes in cancer cells during tumor development are referred to as amino acid metabolic reprogramming.^159^ In addition to glucose metabolism, which provides energy to cancer cells, amino acid metabolism plays an undeniably crucial role in tumor progression. Moreover, amino acids are essential for the biosynthesis of nucleotides, glutathione, glucosamine and polyamines.^160^ They participate in the tricarboxylic acid cycle as metabolites.^161^ Emerging research suggests that alterations in amino acid metabolism are strongly associated with tumor growth, metastasis, and treatment resistance (Table 2).^160^^,^^162^

#### 4.3.1 Glutamine metabolic reprogramming and chemoresistance

Glutamine is a conditionally essential amino acid on which the growth and proliferation of cancer cells heavily depend.^163^ Glutamine is involved in nucleic acid synthesis in cancer cells and provides a nitrogen source for the TCA cycle and lipid biosynthetic pathways. Additionally, glutamine is closely associated with maintaining metabolic redox balance and supporting oxidative metabolic processes.^164^ Augmented glutamine metabolism promotes hypoxia-induced chemoresistance via increasing oxygen consumption in OXPHOS.^165^ In tumors, abnormal regulation of glutamine metabolism can also lead to tumor resistance. Amphiregulin, a ligand for the epidermal growth factor receptor, enhances glutamine metabolism by promoting NADPH production and inhibiting ROS accumulation, thereby supporting cisplatin resistance in human chondrosarcoma.^166^ Another regulator, SLC1A5, a mitochondrial glutamine transporter, is able to block ROS production by inhibiting glutamine-derived GSH synthesis, thereby rendering pancreatic cancer cells resistant to gemcitabine.^167^ TRIM6 overexpression reduces the chemotherapeutic efficacy of cisplatin and paclitaxel by targeting SLC1A5 to inhibit glutamine input and catabolism.^168^ Furthermore, glutamine has been found to activate mTOR signaling. mTOR regulates cellular protein production by modulating translation initiation, ribosome biogenesis, and the biogenesis of several amino acids.^169^ mTORC1 mediates druggable metabolic vulnerabilities by inhibiting autophagy and enhancing resistance to chemotherapy and targeted drugs.^170^ Glutamine regulates mTORC1 via two parallel metabolic pathways. One pathway relies on the enzymatic degradation of glutamine to produce α-ketoglutarate to activate mTORC1, and the other pathway relies on the metabolic bypasses of aspartate synthase and GABA to produce ATP to activate mTORC1 via the inhibition of AMP-activated protein kinase (AMPK) (Fig. 4).^171^

#### 4.3.2 Aspartate and asparagine metabolic reprogramming and chemoresistance

Aspartate (Asp) and asparagine (Asn) are two structurally similar non-essential amino acids. In the progression of cancer drug resistance, they are often synthesized in larger amounts. Within the cell, Asn serves as a precursor to Asp and can be converted into malate, which acts as an intermediate in the tricarboxylic acid cycle and functions as a neuroendocrine neurotransmitter.^172^ Currently, research on Asn is more prevalent compared to Asp.

The inhibition of Asn metabolism has been used to treat ALL for several decades.^173^ L-asparaginase, a commonly used clinical drug, can itself induce drug resistance.^174^ However, L-asparaginase, when combined with other drugs such as doxorubicin and temozolomide, exhibits synergistic effects in cancer treatment.^175^^,^^176^ Recent studies have also revealed additional mechanisms related to Asn. Under conditions of mitochondrial stress, pancreatic cancer cells can activate the GCN2-ATF4 pathway, leading to the constitutive production and release of asparagine through mass action, thereby inducing resistance to mitochondrial inhibitors.^131^ Asn links mitochondrial respiration with ATF4 activity and tumor growth, and it can promote proliferation under metabolic stress, such as rescuing apoptosis in the absence of glutamine.^177^^,^^178^ Elevated asparagine biosynthesis drives metabolic plasticity and resistance to oxidative stress in brain tumor stem cells.^132^ However, it is noteworthy that while Asn promotes the growth of cancer cells, it also has beneficial effects on other cells in the TME. For example, Asn can activate CD8^+^ T cells to kill tumors, and its role in immunotherapy is also worth investigating.^179^

A key function of the TCA cycle in cancer cells is to produce Asp. However, the relationship between Asp and drug resistance is not yet fully understood. Metformin and Phenformin, which inhibit OXPHOS, reduce Asp production and thus suppress resistance to drugs such as EGFR inhibitors.^133^^,^^180^ Mitochondrial aspartate regulates TNF biogenesis, and this area has been studied in autoimmune diseases and may also be a future direction in cancer research.^181^

#### 4.3.3 Branched chain amino acid metabolic reprogramming and chemoresistance

Branched-chain amino acids (BCAAs), including valine, leucine, and isoleucine, are essential amino acids that can serve as nutritional substrates to drive the tricarboxylic acid (TCA) cycle.^182^ BCAAs can also be degraded, providing various metabolites that are involved in tumorigenesis.^183^^,^^184^ The *in vivo* catabolic pathways of BCAAs are primarily regulated by BCAA transaminases (BCATs) and branched-chain alpha-ketoacid dehydrogenase (BCKDH). These key BCAA-related metabolic molecules are considered to be linked to drug resistance. Overexpression of BCAT1, which is expressed on the cell membrane, has been shown to induce resistance to EGFR tyrosine kinase inhibitors (TKIs) and is also considered a key prognostic predictor for cisplatin resistance.^185^^,^^186^ BCAT2, expressed on the mitochondrial membrane, has been found to regulate resistance and ferroptosis.^187^^,^^188^ Additionally, BCKDH expression decreases under Doxorubicin (DOX) treatment, reducing protein translation triggering cell death, ATP insufficiency, and susceptibility to genotoxic stress. Targeting BCKDH can disrupt mitochondrial function and enhance sensitivity to DOX.^189^ Inhibition of branched-chain alpha-keto acid dehydrogenase kinase has been shown to increase the sensitivity of ovarian and breast cancer cells to paclitaxel.^190^

Particularly, there have been studies focusing on individual BCAAs. Under normal conditions, leucine enters the mitochondria for metabolism. Excessive supplementation of leucine can inhibit autophagy via the mTOR pathway, thereby enhancing the sensitivity of AML to doxorubicin.^134^ In breast cancer, LLGL2 can regulate the leucine transporter SLC7A5, promoting leucine uptake and conferring resistance to tamoxifen.^135^ The activation of the Notch signaling pathway in T-ALL leads to an increase in the level of tRNA that transports valine. Restricting valine uptake can inhibit the synthesis of mitochondrial complex I and slow down the progression of T-ALL.^136^

## 5 Downregulated cell death contributes to chemoresistance

Mitochondria play a central role in various forms of cell death, including apoptosis, necroptosis, ferroptosis, and cuproptosis. The mediation process is regulated by a complex protein network. Abnormalities in the signaling pathways associated with mitochondrial-mediated cell death and dysregulated expression of regulatory proteins often occur in tumor cells. In tumor cells, the alterations in cell death signaling lead to reduced sensitivity to cell death and even evasion of drug-induced cell death, thereby contributing to drug resistance.

### 5.1 Mitochondrial pathway of apoptosis in cancer chemoresistance

Apoptosis, one of the earliest defined and most common forms of cell death, refers to a physiological or pathological, active process of cell death triggered by specific signaling pathways.^191^ The mitochondrial pathway, an intrinsic apoptotic pathway, is the most common route for apoptosis. Under the influence of mitochondrial outer-membrane permeabilization (MOMP), mitochondria release cytochrome c, which activates the caspase cascade reaction and initiates apoptosis. This process is effectively regulated by various proteins (such as BAX, BAK, etc.) within the BCL-2 protein family located on the OMM.^192^

**Fig. 5:**
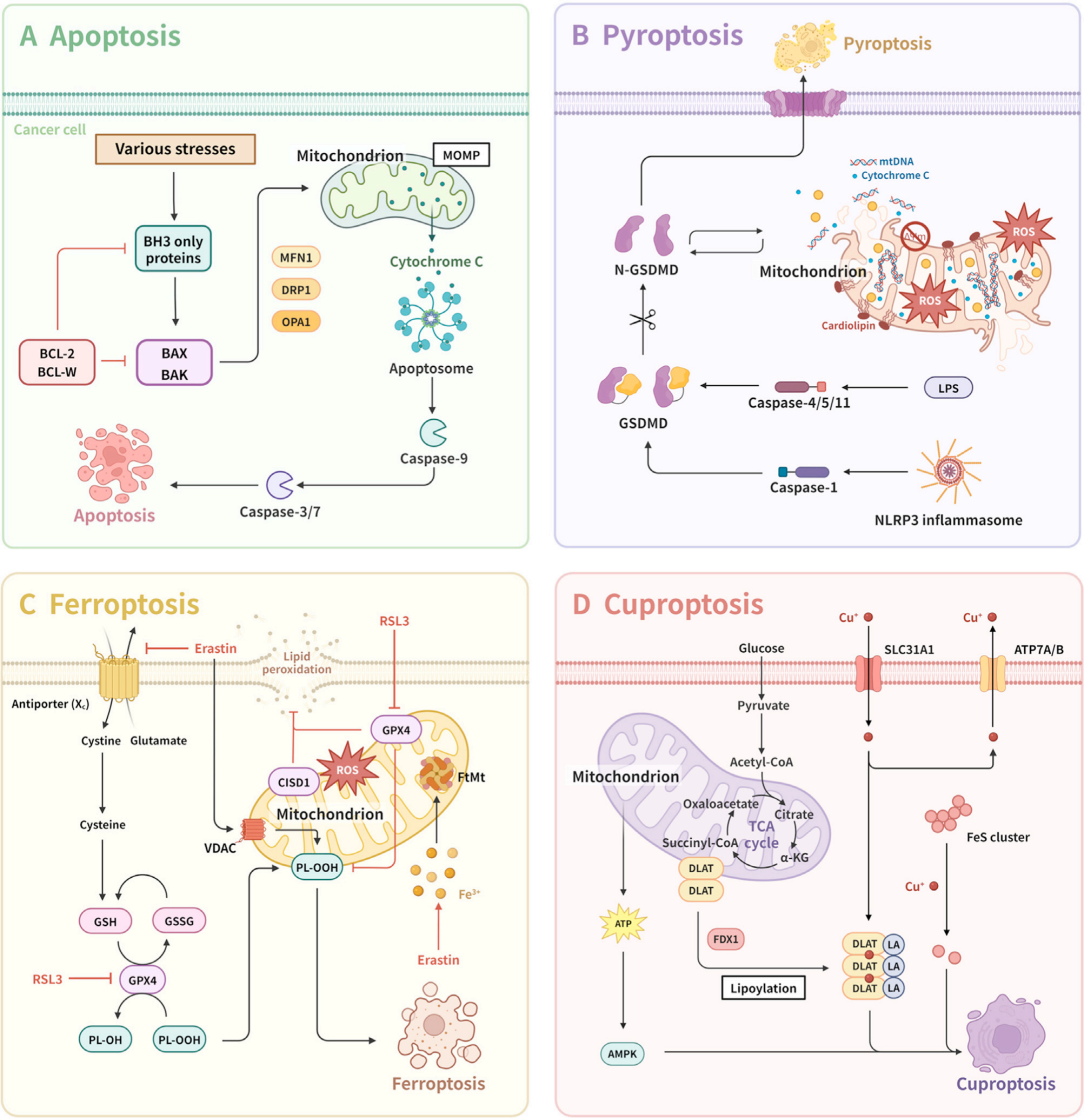
Overview of cell death pathways associated with mitochondria. (A) Apoptosis can be induced intracellular, leading to MOMP, cytochrome c release, apoptotic body formation, and activation of promoter Caspase-9. (B) Caspase-1 lyses GSDMD, releasing fragments that trigger pyroptosis. (C) Ferroptosis can be induced by chemical inhibition of GPX4 by RSL3 or xCT inhibition by erastin, which can disrupt the cell's defense mechanism against lipid peroxidation and induce cell death through large amounts of iron-dependent lipid peroxidation. (D) Cuproptosis is highly correlated with mitochondrial metabolism, and Cu accumulation in the mitochondria leads to the aggregation of fatty acylated DLAT, a protein essential for the mitochondrial tricarboxylic acid (TCA) cycle, thus leading to protein-toxic stress and ultimately to cell death. New attention has been paid to the relationship between multiple cell death patterns associated with mitochondria and tumor chemotherapy resistance, showing potential in cancer therapy. 5-FU, 5-fluorouracil; α-KG, alpha-ketoglutarate; AKR1C3, aldo-keto reductase family 1 member C3; AMPK, AMP-activated protein kinase; Arp2/3, actin-related protein 2/3 complex; Asp, aspartate; Asn, asparagine; ATP, adenosine triphosphate; ATP7A/B, atpase copper transporting alpha/beta; BAK, BCL2-antagonist/killer 1; BAX, BCL-2-associated X protein; BCAA, branched-chain amino acid; BCAT, branched-chain amino acid transaminase; BCKDH, branched-chain alpha-ketoacid dehydrogenase; BCL-2, B-cell lymphoma 2; BCL-W, BCL-2-like protein 2; BH3, BCL-2 homology 3 domain; CISD1, CDGSH iron sulfur domain 1; Cyto c, cytochrome c; DLAT, dihydrolipoamide S-acetyltransferase; DOX, doxorubicin; DRP1, dynamin-related protein 1; ER, endoplasmic reticulum; FAs, fatty acids; FDX1, ferredoxin 1; FIS1, mitochondrial fission 1 protein; GCN2-ATF4, general control nonderepressible 2–activating transcription factor 4; Glu, glucose; GPX4, glutathione peroxidase 4; GSDMD, gasdermin D; GSH, glutathione; HIF-1α, hypoxia-inducible factor-1 alpha; IP3R, inositol 1,4,5-trisphosphate receptor; LC3, Microtubule-associated protein 1 light chain 3; LD, lipid droplet; LPS, lipopolysaccharide; MFN1/2, mitochondrial fusion proteins 1 and 2; MIGA2, mitoguardin 2; miR-522, microRNA-522; MOMP, mitochondrial outer membrane permeabilization; mtDNA, mitochondrial DNA; MTTP, microsomal triglyceride transfer protein; NDP52, nuclear dot protein 52 (CALCOCO2); NLRP3, NOD-like receptor thermal protein domain associated protein 3; OPA1, optic atrophy protein 1; PI(4)P, phosphatidylinositol 4-phosphate; PINK1, PTEN induced putative kinase 1; PLIN1/4, perilipin 1/4; PL-OOH, phospholipid hydroperoxide; PTX, paclitaxel; ROS, reactive oxygen species; SLC, solute carrier family; Treg, regulatory T cells; TUBB3, tubulin beta 3 class III; VPS13, vacuolar protein sorting 13.

In cancer cells, the interplay between mitochondrial dynamics and the BCL-2 protein family significantly influences apoptosis, leading to cancer cell resistance, and molecules such as OPA1, DRP1, and MFN1 play crucial roles.^42^^,^^193^^,^^194^ For instance, OPA1 modulates mitochondrial inner membrane structure during apoptosis, facilitating the release of cytochrome c from mitochondria.^194^ Meanwhile, BAX and BAK enhance DRP1 activity, triggering mitochondrial fission and protecting cancer cells from apoptosis (Fig. 5A).^42^

During conventional cancer chemotherapy, drugs such as taxanes, platinum-based agents, and anthracyclines can induce cell apoptosis to kill cancer cells. Recent studies on resistance to these drugs have revealed key mechanisms. For instance, various classical chemotherapeutic drugs induce cell apoptosis by causing mitochondrial dysfunction and increasing oxidative enzymes, leading to upregulation of ROS, thereby inducing cell apoptosis.^195^ In cancers such as nasopharyngeal carcinoma, colorectal cancer, gastric cancer, and breast cancer, dual-specificity phosphatase 16 (DUSP16) has been found to prevent cisplatin-mediated BAX accumulation and inhibit the mitochondrial apoptosis pathway, thereby promoting resistance of cancer cells to chemotherapy drugs.^196^ Additionally, myo-inositol monophosphatase 2 (IMPA2) affects the p53 pathway by downregulating the expression of Apoptosis-Inducing Factor Mitochondria-Associated 2 (AIFM2), which in turn regulates tumor apoptosis.^197^

Notably, BH3 mimetic drugs, a recently discovered class of drugs targeting the BCL-2 family, inhibits the activity of multiple proteins such as BCL-2 and BCL-W, thus directly inducing cell apoptosis. Clinical studies have found that certain hematologic malignancies such as chronic lymphocytic leukemia (CLL) and AML are particularly sensitive to these drugs and do not develop resistance for a long treatment duration.^198^^,^^199^ However, in solid tumors, stronger resistance to BH3 mimetic drugs has been observed. These tumors can utilize sublethal levels of MOMP and cytosolic cytochrome c to activate heme-regulated inhibitor (HRI) kinase and engage the integrated stress response (ISR), leading to ATF4 synthesis and the acquisition of a drug-tolerant persister phenotype.^200^ This resistance in solid tumors may be due to their relative resistance to apoptosis compared to hematologic malignancies.^201^ Extensive research has explored the resistance mechanisms of BH3 mimetic drugs, including genomics, tumor microenvironment, and metabolic reprogramming. However, their resistance mechanisms remain incompletely understood.^202^

Although independent use of BH3 mimetic drugs may lead to certain drug resistance, combination therapy of BH3 mimetic drugs with other agents holds promising potential. For instance, the combination of the BH3 mimetic drug Venetoclax with mitochondrial autophagy inhibitors may delay resistance to Venetoclax.^9^ Furthermore, through combination therapy, BH3 mimetic drugs may enhance the sensitivity of other types of drugs.^202^ For instance, using BH3 mimetic drugs in combination has been shown to prolong the sensitivity of lung adenocarcinoma to EGFR inhibitors.^203^

### 5.2 Pyroptosis in cancer chemoresistance

Pyroptosis, a newly discovered form of cell death, was first identified in 1992 in murine macrophages infected with Shigella flexneri and officially named in 2001.^204^^,^^205^ The main characteristics of pyroptosis include cell swelling, formation of membrane pores, rupture of the cell membrane, and release of cellular contents. Various cytokines released during the process of pyroptosis can activate immune and inflammatory responses. Caspases 1, 4, 5, and 11 cleave the gasdermin family, especially the pyroptosis effector gasdermin D (GSDMD), playing a crucial role in pyroptosis by causing its pore-forming amino-terminal domain to oligomerize and perforate the plasma membrane. Although previous studies, including single-cell analysis, suggested that mitochondria play a minor role in pyroptosis, recent research has indicated significant mitochondrial involvement in the process of pyroptosis.^206^^,^^207^

Previous research identified that pyroptosis mediated by GSDMD and other gasdermin family members occurs early with mitochondrial damage, and their potential mechanisms and functions are being extensively researched. The impact of mitochondria on cell pyroptosis may not be limited to pathway crosstalk, as there are direct interactions between them.^208^ Miao et al. discovered that the N-terminal pore-forming GSDMD fragment can rapidly disrupt the OMM and IMM, leading to mitochondrial dysfunction, induction of mitochondrial autophagy, loss of transmembrane potential, and the release of mitochondrial proteins and mtDNA, and a series of other reactions.^209^ Additionally, gasdermin A (GSDMA), distinct from GSDMD, preferentially targets the mitochondrial membrane rather than the cell membrane, inducing cell pyroptosis through its action on mitochondria.^210^ Moreover, inducing apoptosis through targeting mitochondrial dysfunction is also achievable. In CRC, IL-17A treatment not only induces mitochondrial dysfunction and stimulates intracellular ROS production but also significantly increases the secretion of inflammatory factors, promoting cancer cell pyroptosis (Fig. 5B).^211^

In the process of cancer development, cell pyroptosis is often considered a double-edged sword.^208^ On one hand, the chronic inflammation associated with cell pyroptosis can provide a microenvironment conducive to tumor initiation and progression. On the other hand, acute and substantial cell pyroptosis can effectively kill cancer cells and activate anti-tumor immunity. Targeting cell pyroptosis has shown certain therapeutic effects on malignant tumors. Recent studies have revealed that targeting cell pyroptosis can not only directly treat malignant tumors but also delay chemotherapy resistance. Su et al. found that integrin β5 upregulates the expression of acid ceramidase (ASAH2), a sphingolipid metabolism enzyme, through STAT3 signaling, thereby reducing the concentration of sphingolipid metabolites and subsequent ROS generation, thus inhibiting chemotherapy-induced cell pyroptosis. Targeting Src signaling can reactivate pyroptosis and reverse resistance.^212^ Additionally, in pancreatic cancer, the expression of gasdermin E (GSDME) is positively correlated with tumor progression, and targeted therapy against GSDME can delay chemotherapy resistance.^213^

Numerous drugs have been reported to treat cancer by inducing pyroptosis, which may also be an important approach to addressing conventional chemoresistance. Decitabine can inhibit DNA methyltransferases, increasing GSDME expression through epigenetic changes, thereby inducing pyroptosis. Combining decitabine with cisplatin liposomes can reduce cisplatin resistance.^214^ Additionally, combining decitabine with pyroptosis-induced photodynamic drugs can reduce the systemic toxicity of chemotherapeutic agents.^215^ Furthermore, numerous nanomedicines are being investigated, with a focus on inducing GSDME expression while increasing caspase-3 levels.^216^, ^217^, ^218^ Some drugs are also being developed to target the caspase-1-GSDMD pathway to induce pyroptosis.^219^

### 5.3 Ferroptosis in cancer chemoresistance

Ferroptosis is a unique mode of programmed cell death, which mainly depends on the accumulation of iron ions and lipid peroxidation in the body. An increasing number of studies have demonstrated that mitochondrial ROS, iron, energy, and other metabolic pathways are closely linked to the ferroptosis process.^220^

Reactive oxygen species (ROS) have a strong correlation with the process of ferroptosis, and mitochondria are one of the main sources of ROS. Studies have shown that the increased mitochondrial ROS can promote ferroptosis, while the removal of mitochondrial ROS can inhibit ferroptosis.^221^ Mitochondrial iron metabolism is also involved in the regulation of ferroptosis. CISD1, a protein containing iron-sulfur clusters located in the outer membrane of mitochondria, and down-regulating the expression of this protein increases iron-mediated lipid peroxidation in mitochondria. These results suggest that iron metabolism in mitochondria plays an important role in lipid oxidation and ferroptosis.^222^ Mitochondrial energy metabolism is also related to the process of ferroptosis. Various regulatory enzymes involved in mitochondrial respiration influence ferroptosis primarily by affecting mitochondrial ROS production, which leads to lipid peroxidation.^223^ In addition, recent studies have revealed the mechanism by which free fatty acids regulate iron death, and phospholipids containing two polyunsaturated fat acyl tails (PL-PUFA2s) are thought to be the driving force of ferroptosis. Mitochondria-targeting antioxidants protect cells from PC-PUFA2-induced mitochondrial ROS, lipid peroxidation, and cell death (Fig. 5C).^224^

As a new cell death mode, ferroptosis provides new therapeutic strategies to overcome cancer chemotherapy resistance. By regulating the key molecules and pathways of ferroptosis, drug-resistant cancer cells can be induced to ferroptosis, thus overcoming the resistance of traditional chemotherapy drugs. Recent studies have shown that the upregulation of mitochondrial protein METTL17 in CRC leads to enhanced resistance of cancer cells to ferroptosis. Knocking down METTL17 disrupts mitochondrial function and energy metabolism, enhances lipid peroxidation and ROS levels in cells and mitochondria, and sensitizes CRC cells to ferroptosis. This effectively inhibits CRC cell proliferation, migration, and invasion.^225^ Similarly, studies have demonstrated that LOXL3, a member of the lysine oxidase (LOX) family, increases its activity in mitochondria through the metabolite kinase AK2 during drug treatment, helping liver cancer cells resist chemotherapy-induced lipid peroxidation and ferroptosis. This resistance renders liver cancer cells tolerant to oxaliplatin and other chemotherapy drugs.^226^ Other studies have shown that energy stress inhibits ferroptosis through AMPK pathway. OXPHOS, an essential pathway for ATP production in mitochondria, plays a crucial role in this process. Energy stress in cancer cells is characterized by a corresponding increase in intracellular ATP consumption and production, and AMPK is an energy sensor. Cancer cells with high levels of AMPK activation are resistant to ferroptosis, potentially triggering drug resistance.^227^ In summary, mitochondria play a central role in regulating iron ion homeostasis, and their abnormal energy metabolism can activate the ferroptosis pathway. At the same time, the damage caused during ferroptosis also impacts mitochondrial function in a feedback loop. Ferroptosis and mitochondrial dysfunction play critical roles in various diseases, providing new and effective strategies for overcoming chemotherapy resistance in cancer.

In fact, a considerable number of drugs targeting ferroptosis have already been developed. Erastin is the commonest drug for inducing ferroptosis. Although the optimized drug piperazine erastin has improved water solubility and metabolic stability to reduce toxicity, its efficacy remains limited.^228^^,^^229^ Further optimization led to the development of imidazole ketone erastin, which shows improved therapeutic effects and can be further optimized when delivered using nanoparticles.^229^^,^^230^ In addition to erastin and its related drugs, as reviewed in detail by Sun et al., GSH synthesis regulators and GPX4 inhibitors also play significant roles in inducing ferroptosis in cancer cells.^231^ Recent advances have shown that targeting carnitine palmitoyltransferase 1A (CPT1A) can induce ferroptosis and synergize with immunotherapy in lung cancer via the CPT1A/c-Myc pathway.^232^ Targeting TRPML1 in AKT-hyperactivated cancers may be a crucial target for inducing ferroptosis.^233^ The application of ferroptosis in cancer treatment continues to be actively explored.^234^

### 5.4 Cuproptosis in chemoresistance

Cuproptosis is a recently identified and distinct form of regulated cell death, first described by Tsvetkov and colleagues in 2022, which is closely associated with mitochondrial metabolism.^235^ When cellular copper (Cu) influx exceeds normal level, Cu may accumulate in mitochondria and subsequently lead to the aggregation of lipoylated dihydrolipoamide S-acetyltransferase (DLAT), a protein essential for mitochondrial TCA cycle. This aggregation induces proteotoxic stress and ultimately results in cell deat.^236^ As a novel mechanism to induce cell death, cuproptosis has brought new attention to overcoming chemoresistance, manifesting great potential in cancer therapies.^237^^,^^238^

While the direct link between copper death and chemoresistance is not particularly close, it has been demonstrated that cuproptosis-related genes and their products do exert a certain degree of influence on various types of cancer.^239^ Pan-cancer studies have suggested that Ferredoxin 1 (FDX1) is significantly associated with the sensitivity to 42 different drugs in various types of cancer.^240^ Additionally, CTR1, a high-affinity copper transporter and copper-transporting ATPases ATP7A and ATP7B, have been found to participate in the efflux of platinum-based drugs, therefore leading to resistance to chemotherapy.^241^ In recent studies, DLAT have also been proved to be closely associated with intracellular ROS elimination and decreased intracellular oxidative stress, ultimately promoting cancer cell survival (Fig. 5D).^242^

Targeting cuproptosis in cancer treatment has been a hotspot in the last two years, as inducing cuproptosis may provide novel therapeutic regimens to overcome chemoresistance. Given that the primary process of cuproptosis occurs in the mitochondria, targeting the mitochondria to induce cuproptosis is one of the most effective methods. Currently, targeting cuproptosis is mainly achieved through nanoparticle delivery systems, which, in addition to directly eliminating cancer cells, often have other functions. A wide range of cancers, such as SCLC, NSCLC and HCC, may be suppressed through overcoming chemoresistance by utilizing nanoparticles.^243^^,^^244^ For example, CuET, a mitochondrial-targeted inducer of cuproptosis, enhances mitochondrial copper exposure, thereby triggering a strong immunogenic cuproptosis in breast cancer cells and inducing M1 macrophage polarization.^245^ CuPEs@PApt is able to activate cuproptosis by addressing both copper overload and glutathione depletion.^246^ Additionally, related drugs can even enhance the response to immunotherapy.^247^ Similarly, nanoparticles such as Cu/TI have also garnered considerable attention.^248^, ^249^, ^250^ In conclusion, targeting cuproptosis still manifest remarkable potential overcoming chemoresistance.

## 6 Chemotherapeutic drug action and mitochondrial mechanistic landscape

Mitochondria are sophisticated organelles that play various roles throughout cancer treatments, especially chemotherapies. In this review, we provide an overall landscape of the relationship between aberrant mitochondrial functions or behaviors and cancer cell chemoresistance (Table 3).

**Table 3:** Drug chemoresistance induced by changes in the mitochondrial landscape.

Drug	Mitochondria-related mechanism	Involved factors	Effects on tumor cells	References
5-fluorouracil	Induces glucose metabolic reprogramming.	HIF-1α	Upregulated HIF-1α levels, mediated by the aberrant activation of PI3K/Akt and Wnt/β-catenin signaling, induce resistance to 5-FU in colorectal cancer CRC.	^122^
	Induces lipid metabolic reprogramming.	LD	Accumulation of LD in cancer cells impairs caspase cascade activation and ER stress response, which can lead to resistance to 5-FU and oxaliplatin therapy.	^91^
	Induces lipid Metabolic Reprogramming.	STAT3	Upregulation of Src-signal transducer and activator of STAT3 inhibits ferroptosis.	^125^
BH3	Resists apoptosis.	HRI	Tumor cells utilize sublethal levels of MOMP and cytosolic cytochrome c to activate HRI kinase and engage the ISR, leading to ATF4 synthesis and the acquisition of a drug-tolerant persister phenotype.	^200^
Carboplatin	Promotes mitochondria-cytoskeleton interaction.	VDAC, β-tubulin, TUBB3	β-tubulin compensate for reduced TUBB3 and maintain DNA repair protein trafficking.	^251^^,^^252^
Cisplatin	Decreases mitophagy.	CRL4, DRP1, Parkin/PINK1, AMPK, MFF	Upregulation of RL4 reduces DRP1 recruitment by inhibiting the phosphorylation of AMPK and MFF, which in turn decreases mitophagy through the Parkin/PINK1 pathway, thereby affecting the resistance of ovarian cancer to cisplatin.	^8^
	Enhances mitochondrial fission.	Spire1C, Arp2/3, DRP1, NRF2	The soft extracellular matrix activates DRP1-mediated mitochondrial fission, leading to an increase in mitochondrial ROS production, which in turn activates the NRF2 antioxidant pathway. Enhanced mitochondrial fission and antioxidant activity contribute to resistance against cisplatin.	^16^
	Enhances mitochondrial fission.	LMP1, AMPK, DRP1	Mitochondrial fission is increased through ROS-mediated AKT activation and subsequent coordinated regulation of the TP53 and NF-κB pathways, promoting autophagy and anti-apoptosis, which regulate the survival of HCC cells.	^18^
	Interrupts the MAM-mediated Ca^2+^ and ROS Signaling.	IP3R, GRP75	GRP75-facilitated MAM formation is enriched under cisplatin-exposure, which may contribute to the increase in [Ca^2+^]m and inhibit pro-apoptotic ROS accumulation, leading to cisplatin resistance.	^66^
	Interrupts the MAM-mediated Ca^2+^ and ROS Signaling.	IP3R, BCL-2	BCL-2 directly targets IP3Rs and prevents pro-apoptotic Ca^2+^ flux, bringing resistance to drugs such as cisplatin.	^67^^,^^68^
	Interrupts the MAM-mediated Ca^2+^ and ROS Signaling.	VDAC1	Enriched VDAC1 in cisplatin-resistant ovarian cancer cells inhibit pro-apoptotic ROS accumulation and maintain cell survival.	^66^
	Promotes mitochondria-lysosome contact.	RAB7	RAB7 regulates resistance to the drug by extracellular vesicle secretion.	^101^
	Induces glucose metabolic reprogramming.	HDAC	Elevated lactate levels can enhance DNA repair and promote cisplatin resistance in cervical cancer cells through the inactivation of HDACs.	^253^
	Induces amino acid metabolic reprogramming.	Amphiregulin, glutamine	Amphiregulin enhances glutamine metabolism by promoting NADPH production and inhibiting ROS accumulation, thereby supporting cisplatin resistance in human chondrosarcoma.	^129^
	Induces amino acid metabolic reprogramming.	TRIM6, SLC1A5	TRIM6 overexpression reduces the chemotherapeutic efficacy of cisplatin and paclitaxel by targeting SLC1A5 to inhibit glutamine input and catabolism.	^254^
	Resists apoptosis.	DUSP16, BAX	DUSP16 prevents cisplatin-mediated BAX accumulation and inhibit the mitochondrial apoptosis pathway, thereby promoting resistance of cancer cells to chemotherapy drugs.	^255^
	Resists cuproptosis.	CTR1, ATP7A, ATP7B	CTR1, ATP7A, and ATP7B have been found to participate in the efflux of platinum-based drugs, therefore leading to resistance to chemotherapy.	^241^
Cytarabine	Promotes mitochondria transfer.	-	Treatment with Cytarabine enhances the capacity of leukemic blasts and leukemia initiating cells toendocytose mitochondria from bone marrow mesenchymal stromal cells, thereby intensifying drug resistance.	^6^
	Decreases mitophagy.	-	Devimistat, by impairing ATP synthesis, can be more effectively targeted by inhibiting mitochondrial fission and autophagy. The combination of Devimistat with Cytarabine and mitoxantrone may treat AML patients who are resistant to Cytarabine.	^10^
Doxorubicin	Promotes mitochondria-LD Contact.	PLIN4	PLIN4 protects the intracellular environment of TNBC cells from the toxic effects of excess FAs and maintains the integrity of LDs.	^95^
	Induces glucose metabolic reprogramming.	ATP, CO2	The inverted pH gradient contributes to MDR by repelling the intake of weakly basic anticancer drugs.	^118^^,^^119^
	Induces amino acid metabolic reprogramming.	BCKDH, BCAA	Loss of BCKDK action in TNBC remodels BCAA flux, reduces protein translation triggering cell death, ATP insufficiency, and susceptibility to genotoxic stress.	^189^
Gefitinib	Promotes mitochondrial fusion.	OPA1	Resistant lung adenocarcinoma cells upregulate OPA1, increase mitochondrial fusion, leading to elongated mitochondria, narrowed cristae, and enhanced ATP production, contributing to resistance against the tyrosine kinase inhibitor gefitinib.	^12^
Gemcitabine	Induces amino acid metabolic reprogramming.	SLC1A5, glutamine	SLC1A5 is able to block ROS production by inhibiting glutamine-derived GSH synthesis, thereby rendering pancreatic cancer cells resistant to gemcitabine.	^256^
	Upregulates the expression of gasdermin E and induces pyroptosis.	GSDME	In pancreatic cancer, the expression of gasdermin E (GSDME) is positively correlated with tumor progression and chemoresistance.	^213^
Methotrexate	Promotes mitochondria transfer.	ICAM-1, TNTs	T-ALL cells adhere to MSCs through ICAM-1, forming TNTs that transfer mitochondria to MSCs, thereby reducing oxidative stress and leading to drug resistance.	^5^
Mitotane	Interrupts the MAM-mediated Ca^2+^ and ROS Signaling	FATE1	FATE1 uncouples the ER and mitochondria in adrenocortical carcinoma cells, thereby decreasing sensitivity to Ca^2+^-dependent apoptosis.	^74^
Oxaliplatin	Induces lipid metabolic reprogramming.	LD	Accumulation of LD in cancer cells impairs caspase cascade activation and ER stress response, which can lead to resistance to 6-FU and oxaliplatin therapy.	^124^
	Resists ferroptosis.	LOXL3	The activity of LOXL3 in mitochondria increases with the aid of AK2 during drug treatment, helping liver cancer cells resist chemotherapy-induced lipid peroxidation and ferroptosis.	^226^
Paclitaxel	Degrades PD-L1 via initiating mitophagy.	ATAD3A, PINK1, PD-L1	PINK1 can recruit PD-L1 to the mitochondria and degrade it through mitophagy. Paclitaxel disrupts the homeostasis of PD-L1 by increasing the expression of ATAD3A, thereby inhibiting PINK1-dependent autophagy.	^11^
	Enhances mitochondrial fatty acid β-oxidation.	CD96, STAT3, OPA1	CD96 expression is associated with poor prognosis, and CD96 activation of the CD155-CD96-Src-Stat3-Opa1 pathway enhances mitochondrial fatty acid β-oxidation, thereby promoting chemoresistance in breast cancer stem cells.	^15^
	Promotes mitochondria-LD Contact.	PGRMC1	PGRMC1 induce dimerization with cytochrome P450, contributes to the colocalization of LD and mitochondria and promotes FAO, which mediates chemoresistance.	^97^
	Induces amino acid metabolic reprogramming.	TRIM6, SLC1A6	TRIM6 overexpression reduces the chemotherapeutic efficacy of cisplatin and paclitaxel by targeting SLC1A6 to inhibit glutamine input and catabolism.	^254^
	Induces amino acid metabolic reprogramming.	BCKDH, BCAA	Inhibition of branched-chain alpha-keto acid dehydrogenase kinase has been shown to increase the sensitivity of ovarian and breast cancer cells to paclitaxel.	^190^
	Resists apoptosis.	IMPA2	IMPA2 affects the p53 pathway by downregulating the expression of AIFM2, which in turn regulates tumor apoptosis.	^197^
Pemetrexed	Induces glucose metabolic reprogramming.	AKR1B10	AKR1B10 regulates LDHA expression and increase lactate-promoted glycolysis, which activates the transcription of the cell cycle-related gene CCNB1, thereby promoting cell survival.	^121^
Sorafenib	Induces glucose metabolic reprogramming.	AKR1C3	AKR1C3 shifts the metabolic profile of HCC from FAO to glycolysis while inhibiting autophagy-dependent degradation of LDs, resulting in LD accumulation.	^96^
Tamoxifen	Blocks mitochondrial fusion.	MFN1, MFN2, OPA1, BAK	Depletion or pharmacological inhibition of MFN1 blocks mitochondrial fusion, restoring BAK oligomerization and cytochrome c release, thereby making resistant cells sensitive to apoptosis and enhancing the therapeutic efficacy of Tamoxifen.	^13^
Temozolomide	Promotes mitochondria transfer.	TNT, Glutamine, Orotic Acid	Mesenchymal stem cells transfer mitochondria to GBM stem cells through TNTs, initiating metabolic reprogramming that includes a transition from glucose to glutamine and an increase in orotate turnover, thereby enhancing the resistance to temozolomide.	^7^
Venetoclax	Downregulates mitophagy.	MFN2, OTUD5, Parkin/PINK1, TP53, BAX, BAK	Upregulation of MFN2 can enhance mitochondria-endoplasmic reticulum interactions and increase mitophagy flux, eliminating mitochondrial damage, thereby enhancing resistance to BH3 mimetics in AML.	^9^
	Promotes mitochondrial fusion.	OPA1, BCL2, MCL1, CLPB	CLPB is upregulated in AML and further induced after venetoclax resistance. CLPB maintains mitochondrial cristae structure through interaction with OPA1, and its loss promotes apoptosis by inducing cristae remodeling and mitochondrial stress responses.	^14^

Abbreviations: AKR1B10, Aldo-keto reductase 1B10; AKR1C3, aldo-keto reductase 1C3; AMPK, Adenosine 5′-monophosphate-activated protein kinase; Arp2/3, actin-related protein 2/3; ATAD3A, ATPase family AAA-domain-containing protein 3; ATP7A, ATPase copper transporting alpha; ATP7B, ATPase copper transporting beta; BAK, BCL-2 killer; BAX, BCL-2-associated X protein; BCAA, branched-chain amino acid; BCKDH, branched-chain alpha-ketoacid dehydrogenase; BCL-2, B-cell lymphoma-2; CD96, cluster of differentiation 96; CLPB, human caseinolytic peptidase B protein homolog; CRL4, Cullin-RING ligase 4; CTR1, copper transport protein 1; DRP1, dynamin-related protein 1; DUSP16, dual specificity phosphatase 16; FATE1, fetal and adult testis expressed 1; GSDME, gasdermin E; GRP75, glucose-regulated protein 75; HDAC, histone deacetylase; HIF-1α, hypoxia inducible factor-1; HRI, heme-regulated inhibitor; ICAM-1, intercellular cell adhesion molecule-1; IMPA2, myo-inositol monophosphatase 2; IP3R, inositol 1,4,5-trisphosphate receptor; LD, lipid droplet; LMP1, latent membrane protein 1; LOXL3, lysyl oxidase like 3; MCL1, myeloid cell leukemia-1; MFF, mitochondrial fission factor; MFN, mitofusin; NRF2, nuclear factor erythroid 2-related factor 2; OPA1, Optic atrophy 1; OTUD5, OTU deubiquitinase; PD-L1, programmed cell death ligand 1; PGRMC1, progesterone receptor membrane component 1; PINK1, PTEN induced putative kinase 1; PLIN4, perilipin 4; RAB7, member RAS oncogene family; SLC1A5, solute carrier family 1 member 5; SLC1A6, solute carrier family 1 member 6; STAT3, signal transducer and activator of transcription 3; TRIM6, tripartite motif-containing protein 6; TNTs, tunneling nanotubes; TUBB3, tubulin beta 3 class III; VDAC, voltage-dependent anion channel.

## 7 Conclusions and further remarks

As multifaceted organelles, the alterations in mitochondria themselves exerts significant impacts on cells. Mitochondria are remodeled in tumors via different mechanisms, including mitochondria transfer, mitophagy, mitochondrial dynamics, and mtDNA defects, influencing cell functions and thereby contributing to chemoresistance. Developing personalized diagnostic and treatment strategies based on the unique mitochondrial behaviors and characteristics in tumor patients may be a significant way to enhance therapeutic success rates. Mitochondria also coordinate with a wide range of other organelles by forming close interaction platforms to regulate cell behaviors and chemoresistance, with the most important being mitochondria-ER contacts. Besides, LDs, lysosomes, cytoskeletons and the Golgi apparatus are also part of the mitochondrial interaction network, exerting unique influences under abnormal regulations. Centering mitochondria-ER contact sites to regulate tumor cell behavior and meanwhile intervening the dysregulation of mitochondrial interactions with other organelles maybe a promising therapeutic strategy. The microstructure changes determine the fate of the cancer cells, and mitochondria mediate this process mainly through two mechanisms. First, mitochondria provide the main stage for metabolic reprogramming, which occurs in nearly all cancer cells for the adaptation to tumor requirements and survival. The other mechanism is the regulation of cell death, as mitochondria serve as a crucial transit point for cell death signals, such as apoptosis, ferroptosis and cuproptosis. Developing metabolism-targeted strategies centered on glutamine, asparate and asparagin metabolism, or ROS to interfere with tumor-specific metabolic adaptations might represent successful strategies. Additionally, exploring synergistic interventions targeting cell death signals to achieve joint regulation may be effective methods to suppress the development of chemoresistance.

The relationship between chemoresistance and mitochondrial functions and behaviors is not a straightforward cause-and-effect interaction. The research on mitochondrial remodeling is currently well-established, but there are still existing gaps in other areas. The description of the interactions between mitochondria and other cellular components, including the Golgi apparatus, cell membrane, and nucleus, still has a long way to go. The specific effects of various metabolites on mitochondria and cells in metabolic reprogramming are not yet fully understood. Research on the various modes of cell death is also incomplete, and clinically, drugs inducing cell death mechanisms such as cuproptosis are not yet in widespread use. Future research needs to address these current gaps by providing comprehensive interconnections, offering new contributions to overcoming chemoresistance and improving patient survival. In conclusion, targeting mitochondria represents a promising way to overcome cancer chemoresistance and opens the door towards more viable strategies for cancer treatment.

## Declaration of competing interest

The authors declare the following financial interests/personal relationships which may be considered as potential competing interests:

Jun Pang reports financial support was provided by the National Natural Science Foundation of China. Yupeng Guan reports financial support was provided by the China Postdoctoral Science Foundation. Jun Pang reports financial support was provided by the Sanming Project of Medicine in Shenzhen. Jun Pang reports financial support was provided by the Research Start-up Fund of Part-time PI, SAHSYSU. Yupeng Guan reports financial support was provided by Guangdong Basic and Applied Basic Research Foundation. Yupeng Guan reports financial support was provided by Postdoctoral Fellowship Program of CPSF. Peng Wu reports financial support was provided by National Undergraduate Training Program for Innovation and Entrepreneurship. If there are other authors, they declare that they have no known competing financial interests or personal relationships that could have appeared to influence the work reported in this paper.
